# BIOMASS YIELD 1 regulates sorghum biomass and grain yield via the shikimate pathway

**DOI:** 10.1093/jxb/eraa275

**Published:** 2020-06-04

**Authors:** Jun Chen, Mengjiao Zhu, Ruixiang Liu, Meijing Zhang, Ya Lv, Yishan Liu, Xin Xiao, Jianhua Yuan, Hongwei Cai

**Affiliations:** 1 Institute of Food Crops, Provincial Key Laboratory of Agrobiology, Jiangsu Academy of Agricultural Sciences, Zhongling Street 50, Nanjing, China; 2 Department of Plant Genetics, Breeding and Seed Science, China Agricultural University; Beijing Key Laboratory of Crop Genetic Improvement; Laboratory of Crop Heterosis and Utilization, MOE; Beijing China; 3 College of Grassland Science and Technology, China Agricultural University, No. 2 Yuanmingyuan West Road, Haidian District, Beijing, China; 4 Forage Crop Research Institute, Japan Grassland Agricultural and Forage Seed Association, 388-5 Higashiakada, Nasushiobara, Tochigi, Japan; 5 The James Hutton Institute, UK

**Keywords:** Biomass yield, flavonoids, metabolic profiling, metabolism, shikimate pathway, sorghum, transcriptome

## Abstract

Biomass and grain yield are key agronomic traits in sorghum (*Sorghum bicolor*); however, the molecular mechanisms that regulate these traits are not well understood. Here, we characterized the *biomass yield 1* (*by1*) mutant, which displays a dramatically altered phenotype that includes reduced plant height, narrow stems, erect and narrow leaves, and abnormal floral organs. Histological analysis suggested that these phenotypic defects are mainly caused by inhibited cell elongation and abnormal floral organ development. Map-based cloning revealed that *BY1* encodes a 3-deoxy-D-*arabino*-heptulosonate-7-phosphate synthase (DAHPS) that catalyses the first step of the shikimate pathway. BY1 was localized in chloroplasts and was ubiquitously distributed in the organs examined, particularly in the roots, stems, leaves, and panicles, which was consistent with its role in biomass production and grain yield. Transcriptome analysis and metabolic profiling revealed that *BY1* was involved in primary metabolism and that it affected the biosynthesis of various secondary metabolites, especially flavonoids. Taken together, these findings demonstrate that BY1 affects biomass and grain yield in sorghum by regulating primary and secondary metabolism via the shikimate pathway. Moreover, our results provide important insights into the relationship between plant development and metabolism.

## Teaser

*BIOMASS YIELD 1* encodes a 3-deoxy-D-*arabino*-heptulosonate-7-phosphate synthase (DAHPS) that affects sorghum biomass and grain yield through regulation of primary and secondary metabolism via the shikimate pathway.

## Introduction

Sorghum (*Sorghum bicolor*) is an annual diploid species (2*n*=2*x*=20) with a small genome (~730 Mb) and it is an agriculturally important short-day C_4_ plant (Paterson *et al.*, 2009). It is one of the world’s five major food crops together with maize (*Zea mays*), rice (*Oryza sativa*), wheat (*Triticum aestivum*), and barley (*Hordeum vulgare*), and is the staple food for more than 500 million people worldwide (Paterson *et al.*, 2009; http://www.fao.org). Sorghum exhibits excellent biomass production due to its strong drought resistance, high photosynthetic efficiency, and good regeneration ability. Hence, it is a crop that is widely cultivated in arid and semi-arid areas, and it is also regarded as a bioenergy crop (Gilbert, 2009; Paterson *et al.*, 2009; Cifuentes *et al.*, 2014; Takaki *et al.*, 2015; Xie and Xu, 2019). Its agricultural importance and the fact that its genome is small and has been sequenced make sorghum an excellent model organism for functional analysis of bioenergy crops (Lawrence and Walbot, 2007; Paterson *et al.*, 2009).

Biomass and grain yield are highly complex agronomic traits that are closely related to the development of various organs and to plant metabolism. The shikimate pathway, representing a bridge between primary and secondary metabolism, is widespread in microorganisms and plants but does not exist in animals (Roberts, 1998; Tzin and Galili, 2010). Secondary metabolites accumulate in plants; in addition to playing important roles throughout growth and development, they improve resistance to invasion by pathogenic microorganisms (Hay, 1996; Kuhn *et al.*, 2011). Flavonoids, a group of secondary metabolites including flavonols, flavones, isoflavones, and anthocyanins, are widely distributed in the flowers, fruits, and leaves of many plants. These metabolites are produced in different tissues, during specific developmental stages, and in response to various environmental factors, and they function in plant defense responses and act as messengers during plant reproduction (Taylor and Grotewold, 2005). Flavonoid biosynthesis occurs via the phenylpropanoid pathway, one of the most well-studied pathways of plant secondary metabolism, which functions downstream of the shikimate pathway (Taylor and Grotewold, 2005; Brunetti *et al.*, 2013).

The shikimate pathway, which is required for the production of aromatic amino acids (AAAs) (Roberts *et al.*, 1998; Yamada *et al.*, 2008; Tzin *et al.*, 2009; Maeda *et al.*, 2011; Ma *et al.*, 2012; Light and Anderson, 2013), involves a seven-step process leading to the formation of chorismate (CHR) via enzymatic reactions involving substrates of phosphoenolpyruvate (PEP) produced by the glycolysis pathway and erythritol-4-phosphate (E4-P) produced by the pentose phosphate pathway (Herrmann, 1995; Tzin and Galili, 2010). CHR, the final product of the shikimate pathway, is a common precursor of various secondary metabolites such as flavonoids, lignin, indole derivatives, and other phenolic compounds (Bentley and Haslam, 1990; Roberts *et al.*, 1998; Ma *et al.*, 2012; Light and Anderson, 2013). DAHPS (3-deoxy-D-*arabino*-heptulosonate-7-phosphate synthase), the first enzyme of the shikimate pathway, plays a central role in linking primary and specialized metabolism (Aharoni and Galili, 2011; Nazmi *et al.*, 2014). Much is known about the function of DAHPS in various microorganisms (Maeda and Dudareva, 2012); however, it shares less than 20% sequence homology between microorganisms and plants and has obvious differences in amino acid composition and properties, suggesting that the functions may differ (Arcuri *et al.*, 2004; Chávez-Béjar, 2008). To date, DAHPS proteins have been identified in several plant species including tobacco (Wang *et al.*, 1991), Arabidopsis (Keith *et al.*, 1991), potato (Zhao and Herrmann, 1992), *Populus trichocarpa* (Tuskan *et al.*, 2006), grapevine (Zhang *et al.*, 2011), tomato (Tzin *et al.*, 2012), and rice (Pagor *et al.*, 2015), but the specific functions and regulatory mechanisms of DAHPS in plants are not well understood.

In this study, we characterized the sorghum mutant *biomass yield 1* (*by1*), which exhibits reduced plant height, narrow stems, narrow and erect leaves, and abnormal floral organs. Map-based cloning revealed that *BY1* encodes a DAHPS. Functional analysis revealed that *BY1* is constitutively expressed in sorghum and that it participates in the first step of the shikimate pathway. Transcriptome analysis and metabolic profiling suggested that *BY1* influences the cell cycle and metabolite levels. Our observations suggest that BY1 is a DAHPS that influences biomass production and grain yield in sorghum by modulating primary and secondary metabolism via the shikimate pathway.

## Materials and methods

### Plant materials and growth conditions

In this study we used three *Sorghum bicolor* (Moench) parent lines, namely BTx623, the *biomass yield 1* (*by1*) mutant, and Shangzhuang (a local broom sorghum variety in Beijing) together with two mapping populations, namely *by1* × Shangzhuang and BTx623 × *by1*. The *by1* mutant was isolated from an ethyl-methanesulfonate (EMS) mutant population developed by our group in the BTx623 background, an elite sorghum inbred line with an available genome sequence. In brief, about 4000 healthy BTx623 seeds were treated with 0.25% EMS and the mutant lines were selected from a segregating M_2_ population of selfed M_1_ plants according to various traits including tillering, panicle length, grain weight, and plant height. The *by1* mutant was backcrossed with BTx623 twice to eliminate interference from other invalid mutation sites, and was then used for subsequent analyses of phenotype, mapping, expression, transcriptome, and metabonomics. An F_2_ mapping population was constructed by crossing *by1* with Shangzhuang. All sorghum plants were grown in 2015–2019 at the Shangzhuang Experimental Station of the China Agricultural University, Beijing, China, in the summer and at another experimental field in Sanya, Hainan Province, in the winter. Plants were grown in rows at 70-cm spacing and each row contained 10 plants with 25-cm spacing. Paper bags were used to cover the panicles of the plants before flowering to prevent cross-pollination.

### Phenotypic analysis

Twenty plants each of BTx623 and *by1* were collected and the following agronomic traits were determined: plant height, leaf length, leaf width, internode length, internode diameter, seed-setting rate, spike length, and 1000-grain weight.

### Microscopy

For histological analysis, fresh internode tissue from the middle portions of the panicle neck internodes were collected from *by1* and BTx623 plants at the heading stage and fixed in 3.7% FAA solution (3.7% formaldehyde, 70% ethanol, and 5% glacial acetic acid). After incubation with shaking at 4 °C overnight, the samples were subjected to a series of dehydration and infiltration steps. The samples were embedded in paraffin, cut into 8-µm sections using a Leica RM2265 microtome, stained with 1% Safranin O and 1% Fast Green FCF, and imaged under a BX51 microscope (Olympus).

For SEM, flagleaf tissue was collected from *by1* and BTx623 plants at the heading stage, cut into small pieces, and immediately fixed in 2.5% glutaraldehyde in 0.2 M phosphate buffer (pH 7.0). The samples were incubated at 4 °C overnight, washed with the same phosphate buffer, post-fixed in 1% OsO_4_, and dehydrated through a graded ethanol series (30–100%). The samples were then critical-point dried, coated with gold, and observed under a Hitachi S-3400N SEM. Intact, round pollen grains were considered to be fully developed, whilst those that appeared folded were counted as defective. The proportion of pollen with good integrity relative to the total number within a field of vision was calculated. The cell lengths and widths were measured using the ImageJ software.

### Map-based cloning of *BY1*

Map-based cloning of *BY1* was performed using an F_2_ mapping population generated by crossing the *by1* mutant with the sorghum inbred line Shangzhuang. Total genomic DNA was extracted from fresh leaves using the CTAB (cetyltrimethylammonium bromide) method (Murray and Thompson, 1980), with minor modifications. Approximately 110 pairs of published SSR markers (Li *et al.*, 2009; Yonemaru *et al.*, 2009) with polymorphism between the parents were used for bulked segregant analysis (BSA; Michelmore *et al.*, 1991) to identify the linkage markers. A total of 269 F_2_ plants were used for mapping the *BY1* locus. Fine-mapping was achieved by increasing the numbers of SSR makers in 2821 F_2_ plants. A list of the primers used for mapping and for all other analyses is given in Supplementary Table S3 at *JXB* online.

### Phylogenetic analysis

Using the BY1 protein sequence as a query, we searched the Phytozome (https://phytozome.jgi.doe.gov/pz/portal.html) and NCBI (https://www.ncbi.nlm.nih.gov) databases. An *E*-value cut-off of 0.0 was used to identify BY1-like proteins in other species. CLUSTAL W was used to align all the BY1-like protein sequences (Thompson *et al.*, 1994). The WebLogo software was used for motif analysis (Crooks *et al.*, 2004) and the MEGA 5.0 software was used for construction of the phylogenetic tree via the neighbor-joining method (Tamura *et al.*, 2011).

### Genetic transformation

Based on homology analysis, Loc_Os07g42960 in rice was identified as the homologous gene of sorghum *by1*. To generate the knock-out vector for Loc_Os07g42960, two gRNAs (*Osby1-1* and *Osby1-2*) targeting two different sites in the first exon of Loc_Os07g42960 were designed using CRISPR-P (http://crispr.hzau.edu.cn/CRISPR2/). *Osby1-1* and *Osby1-2* were introduced into p*YLCRISPR/Cas9-MH*, a CRISPR/Cas9 binary vector. The CRISPR/Cas9 vector containing the *Osby1-1* and *Osby1-2* targets and an empty CRISPR/Cas9 vector were introduced into ZH11, a *japonica* rice variety commonly used for genetic transformation, via *Agrobacterium tumefaciens* EHA105-mediated transformation.

### Subcellular localization of BY1

The p*35S::BY1-GFP* and p*35S::by1-GFP* vectors were constructed and used for subcellular localization analysis. The p*35S::BY1-GFP* vector contained the full-length cDNA sequence of *BY1* fused with green fluorescent protein (GFP), and p*35S::by1-GFP* contained the full-length cDNA sequence of *BY1* from the *by1* mutant fused with GFP; both genes were driven by the 35S promoter. The vectors were introduced into maize leaf protoplasts as described by Liu *et al.*, (2017), with minor modifications. GFP fluorescent signals were visualized under a confocal laser-scanning microscope (LSM710, Zeiss).

### Enzyme activity assays of DAHPS

The enzyme activity of DAHPS was determined using a Plant 3-deoxy-D-arabinoheptulosonate-7-phosphatesynthase (DAHPS) ELISA Kit (Shenzhen Ziker Biological Co., Ltd). Young leaves from wild-type (BTx623) and *by1* plants at the 6-leaf stage were ground on ice to extract total enzyme. The test samples, standard samples, and the HRP (horseradish peroxidase)-labeled test antibody were added to micropores coated with a plant anti-DAHPS antibody, incubated, and washed thoroughly. The substrate TMB (3´,3´,5´,5´-tetramethylbenzidine) was used to develop the color, after which the absorbance was measured at 450 nm using an Enzyme Standard Instrument (Power Wave XS2, BioTek), and the DAHPS activity was calculated.

### RNA extraction and RT-qPCR

Total RNA was extracted and purified from various tissues of wild-type and *by1* mutant plants at the booting stage, namely the roots, nodes, stems, young leaves (leaf blade), leaf sheaths, and young panicles, using Trizol reagent (Invitrogen) and an RNA-Clean Kit (Tiangen, DP412). First-strand cDNA was generated from ~2.5 μg total purified RNA by reverse-transcription using oligo(dT)_18_ primers (TaKaRa) and M-MLV Reverse Transcriptase (Promega, M1701). RT-qPCR was performed using a CFX96 Real-Time System (Bio-Rad), and the sorghum *PP2A* gene SORBI_3004G092500 was used as an internal control (Palakolanu *et al.*, 2016). At least three biological and three technical replicates were used for all sample, and all expression data were generated using the relative quantification method (Livak and Schmittgen, 2001).

### Transcriptome and metabolic profiling

Transcriptome analysis was performed by Annoroad Gene Technology (Beijing) Co., Ltd (http://www.annoroad.com/). Young leaves (~3 cm at the tip of the seventh leaf) were harvested from wild-type and *by1* plants at the 6–7-leaf stage, when the sixth leaf was about 50% unfolded and about 20% of the seventh leaf had emerged. Samples from five plants were ground into a fine powder in liquid nitrogen and pooled as a single biological repeat, and three biological repeats were performed for both wild-type and *by1* plants. Total RNA was extracted and purified using Trizol reagent (Invitrogen) and a RNAClean Kit (Tiangen, DP412). The libraries were constructed and sequenced using the Illumina HiSeq2000 platform. For each sample, 6 GB of RNA-seq data were obtained (~8–9× coverage of the sorghum genome). Low-quality reads (reads containing sequencing Ns>10%), short reads (Q<10 nt), and adaptor sequences were removed by filtering the raw sequences. All high-quality, clean reads were mapped to the sorghum reference genome (BTx623) using the TopHat2 software (https://ccb.jhu.edu/software/tophat/). The resulting RNA-seq data were normalized as the number of RPKM (reads per kilobase per million mapped reads) using the Cuffquant and Cuffnorm software (https://cole-trapnell-lab.github.io/cufflinks/) and used for subsequent transcriptome analysis. Significant differentially expressed genes (DEGs) between the wild-type and *by1* were identified using the Cuffiinks software (Trapnell *et al.*, 2010). Genes with at least a 2-fold change in expression and a false-discovery rate of <0.05% were defined as differentially expressed.

Metabolome analysis was performed by Wuhan Maiwei Biotechnology Co., Ltd (https://www.metware.cn/Home11.html) using a widely targeted metabolome method. All samples used for metabolome analysis were at the same stage and position as those used for the transcriptome analysis. Metabolites were quantified using a liquid chromatography-electrospray ionization-tandem mass spectrometry (LC-ESI-MS/MS) system (Zhu *et al.*, 2018). Each 100 mg sample of dry powdered tissue was extracted overnight at 4 °C with 1 ml 70% aqueous methanol solution containing 0.1 mg l^–1^ lidocaine. A scheduled multiple reaction monitoring method was used to quantify the metabolites (Chen *et al.*, 2013).

### Statistical analysis

The two-tailed Student’s *t*-tests were performed on all the data using the SPSS software.

### Accession numbers

Gene sequences of *by1* have been deposited in GenBank with the accession number MN832739 (*by1* mutant).

## Results

### Characterization of the *by1* mutant

**Fig. 1.**
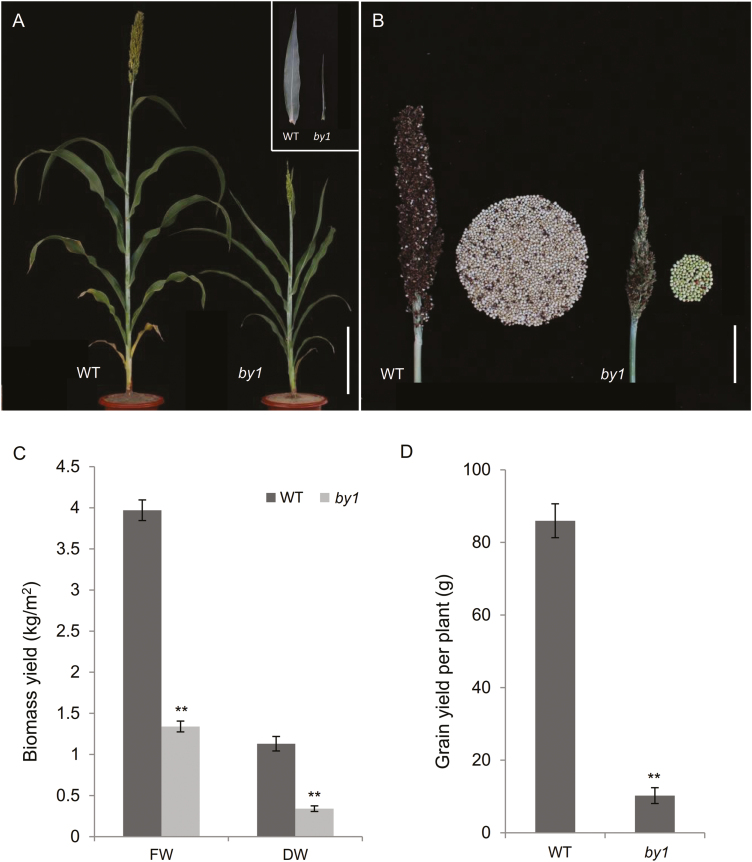
Biomass production and grain yield in wild-type sorghum and the *by1* mutant. (A) Phenotypes of the wild-type (WT) and *by1* plants at the flowering stage. Scale bar is 20 cm. (B) Phenotypes of panicles at maturity. Scale bar is 10 cm. (C) Biomass yields in terms of fresh and dry weight. (D) Gain yield (DW). Data are means (±SD), *n*=20. Significant differences were determined using two-tailed Student’s *t*-test: *P<0.05, **P<0.01.

We constructed a sorghum mutant library via EMS mutagenesis of a BTx623 population (an elite sorghum inbred line with an available genome sequence) and identified the *biomass yield 1* (*by1*) mutant. At the flowering stage, *by1* plants exhibited lower height, narrower stems, and narrower leaves compared to the wild-type (WT), which resulted in a significant decrease in biomass production (Fig. 1A, C). At maturity, *by1* plants exhibited a shorter panicle length, lower seed-setting rate, shorter grain length, narrower grain width, and lower 1000-grain weight than the WT, which led to a significant decrease in grain yield per plant (Fig. 1B, D, Supplementary Fig. S1E, F, Supplementary Table S1).

### Histological analysis

**Fig. 2.**
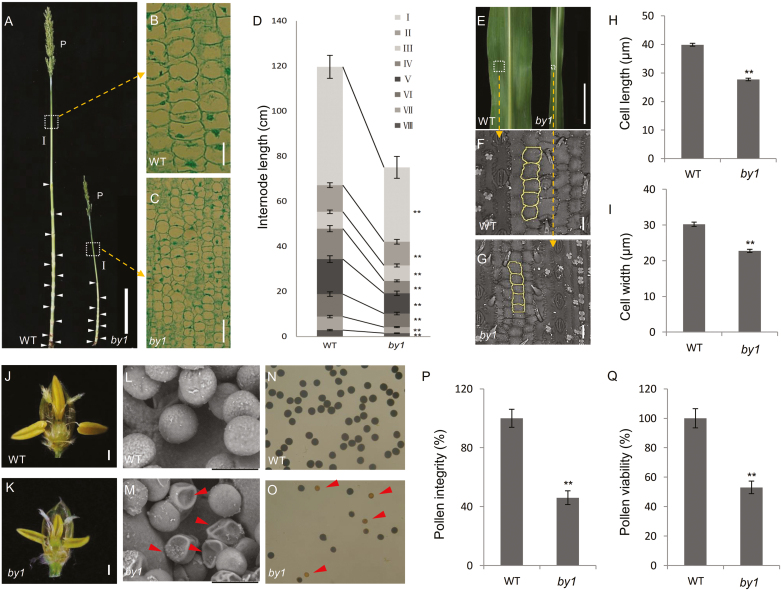
Histological analysis of wild-type sorghum and the *by1* mutant. (A) Stems of the wild-type (WT) and *by1*. White arrowheads indicate nodes. Scale bar is 20 cm. (B, C) Longitudinal sections of the panicle neck internodes of the WT and *by1*. Scale bars are 50 µm. (D) Comparison of internode lengths between the WT and *by1*. (E) Leaves of the WT and *by1* (scale bar is 10 cm), and (F, G) corresponding SEM images, with cell morphologies highlighted (scale bars are 30 µm). (H) Leaf cell lengths and (I) cell widths in the WT and *by1*. (J, K) Images of fertile florets in the WT and *by1*. Scale bars are 1 mm. (L, M) Images of pollen grains of the WT and *by1*. Scale bars are 50 µm. (N, O) Staining for pollen viability in the WT and *by1*. (P) Pollen integrity, as determined from images in (L, M). (Q) Pollen viability, as determined from images in (N, O). Data are means (±SD), *n*=10, except *n*=20 in (D). Significant differences were determined using two-tailed Student’s *t*-test: *P<0.05, **P<0.01.

We examined the length and diameter of each internode at the flowering stage and found that all *by1* internodes were shorter and smaller than those of the WT (Fig. 2A, D). To examine the reasons for the decreased plant height and thinner stems in the mutant, we performed histological analysis of the middle portions of the panicle neck internodes. Although the internode cells of both the WT and *by1* were well developed, the cell volume was significantly reduced in the mutant (Fig. 2B, C, Supplementary Fig. S1A–D). Compared to the WT, the leaf length and width were significantly reduced in *by1*. We performed SEM and paraffin sectioning to examine leaf-blade and leaf-vein cells, respectively, and found that both were well developed in both the genotypes; however, the cell lengths and widths were significantly reduced in *by1*, resulting in a smaller cell volume compared to the WT (Fig. 2E–I; Supplementary Fig. S2).

In addition to the differences in plant height and in the leaves, the grain yield was significantly reduced in *by1*. Comparing the young panicles of WT and *by1* plants before flowering, we found severe developmental defects in the mutant, especially at the tips of panicles (Supplementary Fig. S3). We also compared the phenotypes of fertile florets at the flowering stage, and found that all those of the WT were well developed and their anthers were golden-yellow and plump, whereas those of *by1* showed various developmental defects and their anthers were light-yellow, thin, and small (Fig. 2J, K; Supplementary Fig. S4). Under SEM, the pollen grains of *by1* were found to be smaller than those of the WT, and ~50% of them appeared crumpled (Fig. 2L, M, P). When stained with 1% I_2_-KI solution, almost all the WT pollen appeared dark brown, indicating viability, whereas only 50% of *by1* pollen was stained (Fig. 2N, O, Q). Overall, these results suggested that the phenotypic changes in the *by1* mutant were the result of reduced cell elongation and abnormal development of the flower organs.

### Map-based cloning of *BY1*

To determine the genetic characteristics associated with the altered phenotypes, we backcrossed *by1* plants with the WT, BTx623. All F_1_ plants showed WT phenotypes, whilst ~25% of plants in the F_*2*_ population exhibited *by1* phenotypes (83 *by1* plants and 241 WT plants; *χ*^2^_c_ =0.037, *χ*^2^_0.05,1_=3.84). This segregation was very close to the hypothesized ratio of 3:1, indicating that the altered phenotypes of *by1* were caused by a single, recessive Mendelian gene.

**Fig. 3.**
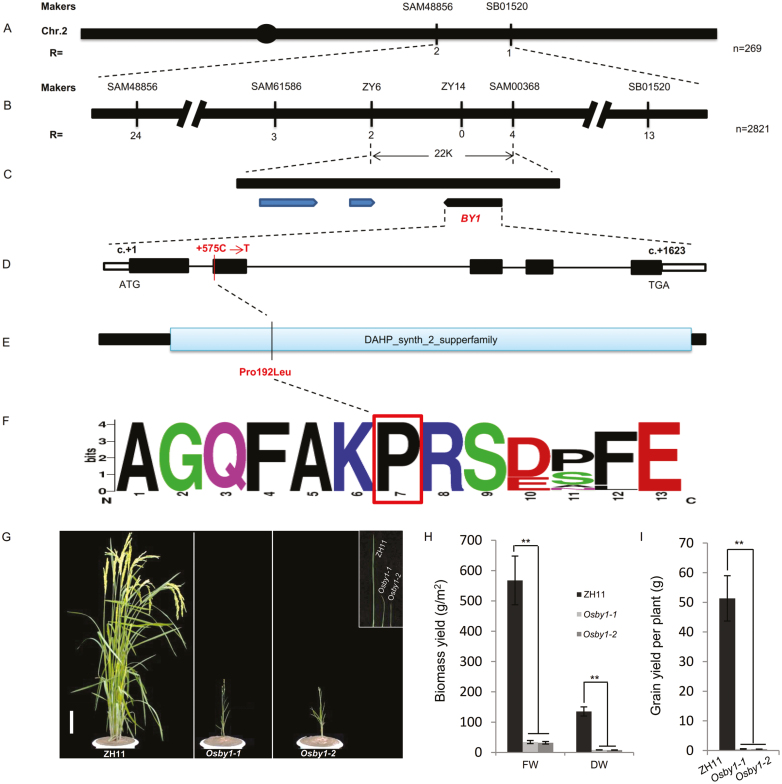
Map-based cloning of sorghum *BY1*. (A) Rough and (B) fine-mapping of *BY1*, where *n* is the total number of plants used for recombinant screening and mapping, and *R* is the number of recombinant individuals identified by screening recombinants between flanking markers. (C) Genes in the fine-mapping region of *BY1*. (D) The structure of *BY1* and the *by1* mutation site. The unfilled boxes at the left and right ends represent the 5´-UTR and 3´-UTR, respectively. The black boxes represent exons and the lines between them represent introns. The red line and text at the second exon indicate the position and form of base mutation in the *by1* mutant. (E) The amino acid structure of *BY1* and the mutation site in *by1*. The blue box represents the functional domain region, and indicates the position and form of the amino acids in the *by1* mutant. (F) Conservation analysis of the amino acid substitution region and frequency in homologous plant genes. The red box indicates the position of the amino acid substitution in *by1*. (G–I) The *japonica* rice cultivar ZH11 was gene-edited using CRISPR/Cas9. *Osby1-1* is a line containing target 1 and *Osby1-2* is a line containing target 2. (G) Phenotypes of the lines. The scale bar is 5 cm. (H) Biomass yield and (I) grain yield of the lines (both DW). Data are means (±SD), *n*=10. Significant differences compared with the ZH11 wild-type were determined using two-tailed Student’s *t*-test: **P<0.01.

To clone *BY1*, we generated another F_2_ population derived from a cross between *by1* and Shangzhuang, a local broom sorghum variety. Using map-based cloning, we roughly mapped the *BY1* gene to the end of the long arm of chromosome 2, in a region between the simple-sequence repeat (SSR) markers SAM48855 and SB01520. To fine-map the *BY1* locus, we then used another 2821 additional individuals from the same F_2_ population for recombinant screening, and the *BY1* gene was further delimited to a 22-kb region between the SSR markers ZY6 and ZY21. Three predicted ORFs were located in this region according to the sorghum genome sequence database (https://phytozome.jgi.doe.gov/pz/portal.html) (Fig. 3A–C). Sequencing of the mapped genomic regions in *by1* and the WT revealed only one single-nucleotide polymorphism (SNP). A transformation from C to T at position +575 (from the ‘A’ in the start codon of *BY1*) in exon 2 of Sobic.002G379600 caused a Pro192Leu amino acid substitution in the mutant (Fig. 3D, E), indicating that this was the the potential candidate gene for *BY1*.

To verify that Sobic.002G379600 was *BY1*, we compared its sequence in 164 lines of the sorghum germplasm collected worldwide, and found that they all showed the same genotype as the WT at the site of the *by1* mutation (Supplementary Data S1). We randomly selected and sequenced 20 recessive plants from the *by1* × Shangzhuang F_2_ population And found that a;; of them contained the same mutation site as *by1* (Supplementary Fig. S5). These results suggested that the *by1* mutation was induced by EMS and that it may not exist in natural sorghum accessions. Furthermore, phylogenetic analysis showed that *BY1* is widely conserved in plants and shares the closest relationships with genes from other Poaceae, including *Panicum virgatum*, *Pa. hallii*, *Setaria viridis*, *Se. italic*, *Zea mays*, *Oropetium thomaeum*, *O. sativa*, *Brachypodium stacei*, and *B. distachyon* (Supplementary Fig. S6). Importantly, multiple-sequence alignment and motif analysis revealed that the 192Pro amino acid is conserved in higher plants (Fig. 3F). These results implied that the Pro192Leu amino acid substitution in *by1* may be responsible for its growth defects.

Since it is difficult to perform genetic transformation in sorghum and given that *BY1* is highly conserved between sorghum and rice, we used the *japonica* rice variety ZH11 (commonly used in genetic studies) as the recipient material for transformation. We generated a knock-out construct using the binary vector p*YLCRISPR/Cas9-MH* containing two different gRNAs, named *OsBY1-1* and *OsBY1-2*. We introduced this construct into ZH11 to knock out Loc_Os07g42960, the homologous gene of sorghum *by1* in rice, and obtained 29 successfully edited plants. These plants exhibited seven different genotypes, as revealed by sequencing. As expected, all of the plants showed dwarfing, narrow leaves, poor spike development, and possessed significantly reduced biomass and grain yield compared to the WT, making them highly similar to the phenotypes of the sorghum *by1* mutant (Fig. 3G–I, Supplementary Fig. S7).

These results suggested that Sobic.002G379600 is *BY1*, which regulates biomass and grain yield in sorghum, and that the function of *BY1* is conserved between sorghum and rice.

### Transcriptional and functional characterization of *BY1*

To obtain the full-length *BY1* sequence, we used the RACE technique. The cDNA of WT *BY1* is 2039 bp long, with a 77-bp 5′-untranslated region, a 1623-bp ORF, and a 339-bp 3′-untranslated region. The gene contains five exons and four introns and encodes a 540 amino-acid protein (Supplementary Fig. S8).

**Fig. 4.**
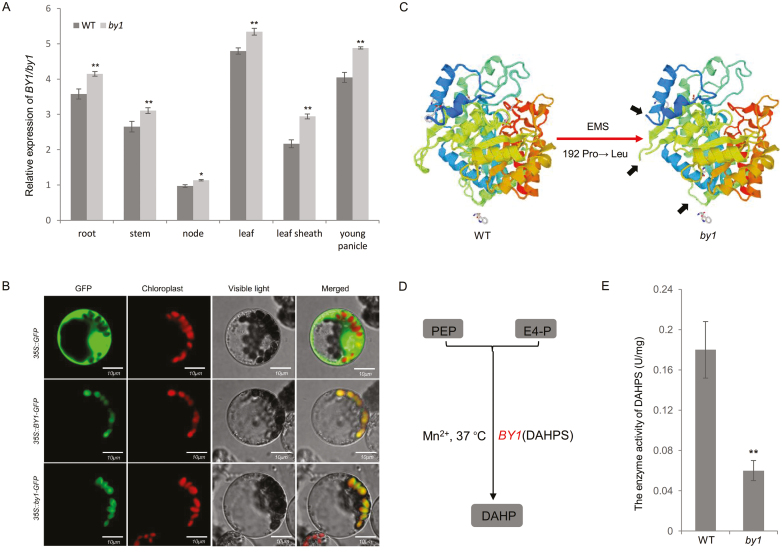
Transcriptional and functional characterization of *BY1.* (A) Relative expression of *BY1* in different tissues of the sorghum wild-type (WT) and the *by1* mutant. (B) Subcellular localization of the BY1 and by1 proteins in maize protoplasts. 35S*::GFP* is the control (top row), 35S*::BY1-GFP* fused with the full-length sequence of *BY1* is the WT gene fusion (middle row), and 35S*::by1-GFP* fused with the full-length sequence of *by1* is the mutant gene fusion (bottom row). Scale bars are 10 μm. (C) Comparison of the tertiary structure of the BY1 protein in the WT with by1 in the mutant. The structures were predicted using SWISS-MODEL (https://swissmodel.expasy.org/). Black arrows indicate the obvious structural alterations between the WT and by1 due to the conversion of the 192nd amino acid from Pro to Leu. (D) Reaction scheme for 3-deoxy-D-*arabino*-heptulosonate-7-phosphate synthase (DAHPS, BY1). PEP, phosphoenolpyruvate; E4-P, erythrose-4-phospate. (E) Enzyme activity of DAHPS (BY1) in the WT and the *by1* mutant. Data are means (±SD), n=3. Significant differences were determined using two-tailed Student’s *t*-test: *P<0.05, **P<0.01.

To investigate the expression patterns of *BY1*, we performed a RT-qPCR analysis and found that it was constitutively expressed in the roots, stems, nodes, leaves, leaf sheaths, and young panicles. Compared to the WT, *BY1* was slightly up-regulated in all these tissues in the *by1* mutant (Fig. 4A). We examined subcellular localization by fusing the coding sequence of *BY1* in the WT and *by1* to *GFP* to generate the fusion constructs p35S*::BY1-GFP* and p35S*::by1-GFP*, respectively, and transferred these into maize protoplasts. The BY1 fusion proteins were specifically localized to chloroplasts, and the *by1* mutation did not affect this (Fig. 4B).

Since the mutation site occurred in the conservative domain of *BY1* and the physical and chemical properties of Pro and Leu are quite different, we hypothesized that the mutation might result in a structural change in the BY1 protein. To test this, we used SWISS-MODEL (https://swissmodel.expasy.org/) to examine the tertiary structure and found that it was indeed significantly altered when the 192nd amino acid was changed from Pro to Leu (Fig. 4C; Supplementary Fig. S15).

Protein BLAST analysis of the GenBank database (http://www.ncbi.nlm.nih.gov/BLAST/) and Pfam analysis showed that *BY1* encodes a DAHPS, which catalyses the formation of 3-deoxy-D-*arabino*-heptulosonate 7-phosphate (DAHP) from phosphoenolpyruvate (PEP) and erythrose-4-phospate (E4-P) (Fig. 4D). To determine whether the mutation led to loss of function, we assayed the enzyme activity of BY1 with PEP and E4-P. Compared to the WT, the enzyme activity of the *by1* mutant was significantly reduced, implying that BY1 lost its normal function as a result of the mutation (Fig. 4E).

### Transcriptome analysis and metabolic profiling

To elucidate the molecular mechanism underlying how *BY1* influences development in sorghum, we performed transcriptome analysis and metabolic profiling using the leaves of WT and *by1* plants at the 6–7-leaf stage, when the phenotypes of the plants were markedly different. We examined three biological replicates, with each sample consisting of five individual leaves of different plants at the same age and position.

**Fig. 5.**
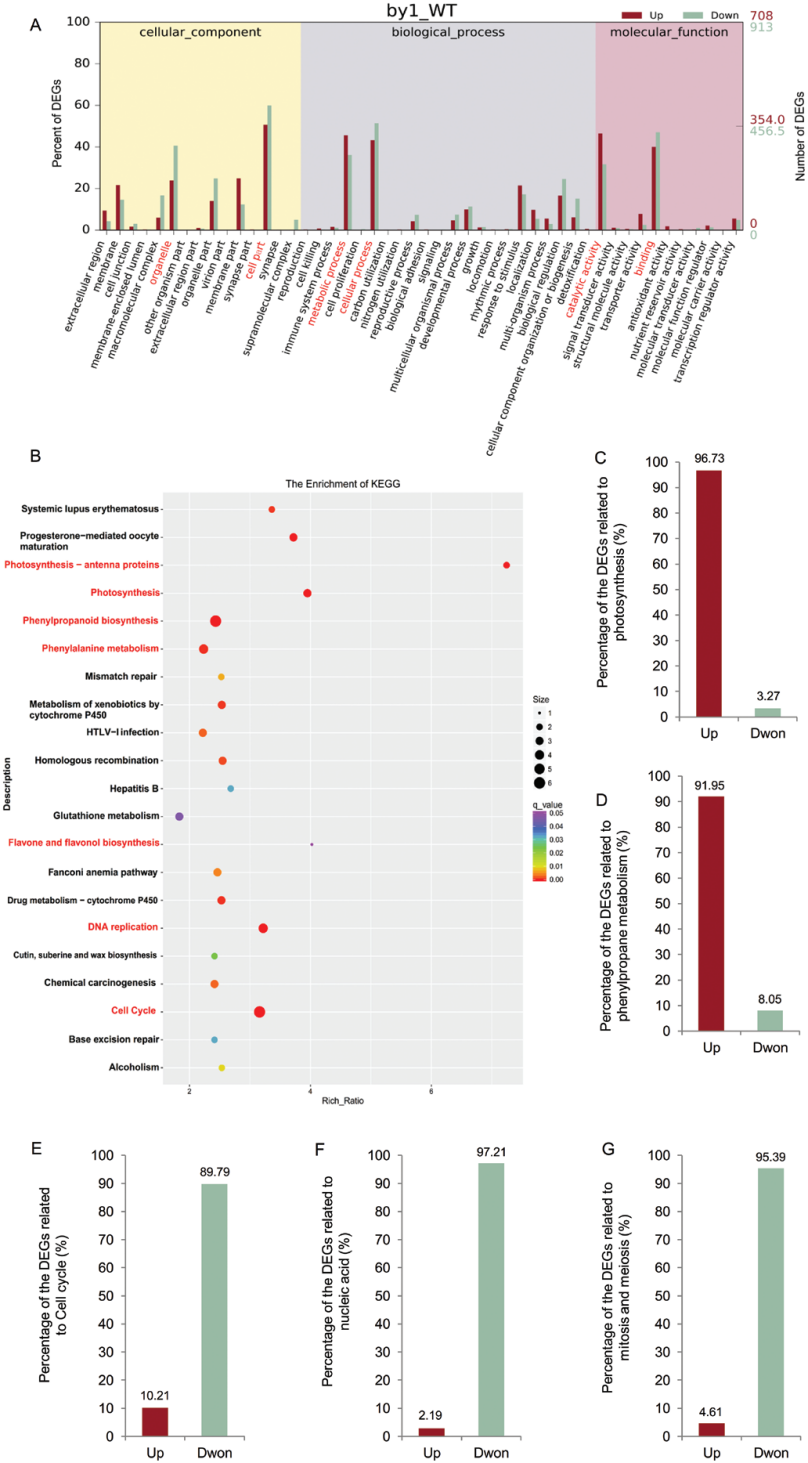
Transcriptome analysis of sorghum *BY1*. (A) Gene ontology (GO) analysis of differentially expressed genes (DEGs). The numbers of up- and down-regulated DEGs in each functional pathway are indicated on the right axis, and their percentages are indicated on the left axis. (B) *q*-values of individual groups of enriched KEGG pathways. Each point represents the degree of enrichment of the KEGG category according to the color scale, whilst the number of DEGs is indicated by the size of the point. (C–G) Percentages of up- and down-regulated DEGs related to (C) photosynthesis, (D) phenylpropane metabolism, (E) the cell cycle, (F) nucleic acids, and (G) mitosis and meiosis.

We defined significantly differentially expressed genes (DEGs) as those with a fold-change ≥2 or ≤0.5, and *q*<0.05. Compared to the WT, a total of 1785 DEGs were detected in all three biological replicates in *by1*, of which 851 were up-regulated and 934 were down-regulated (Supplementary Fig. S9A, B, Supplementary Data S2). Using the hierarchical clustering method, we clustered the DEGs into six subclasses based on their expression levels (Supplementary Fig. S9C). We then performed gene ontology (GO) analysis and divided the DEGs into the categories ‘biological process’ (BP), ‘molecular function’ (MF), and ‘cellular component’ (CC). The DEGs were enriched in many different GO terms, especially ‘cell part’ and ‘organelle’ in the CC category, ‘metabolic process’ and ‘cellular process’ in the BP category, and ‘catalytic activity’ and ‘binding’ in the MF category (Fig. 5A).

GO enrichment and KEGG (Kyoto Encyclopedia of Genes and Genomes) analysis demonstrated that *BY1* influenced various biological pathways, including photosynthesis, the cell cycle and division, and primary and secondary metabolism (Fig. 5B, Supplementary Fig. S9D, E). We further explored the expression patterns of DEGs related to photosynthesis, the cell cycle, phenylpropane metabolism, nucleic acid, and mitosis and meiosis. Interestingly, almost all DEGs related to energy biosynthesis pathways, such as photosynthesis and phenylpropane metabolism, were significantly up-regulated in *by1* compared with the WT (Fig. 5C, D, Supplementary Fig. S10A, B). In contrast, almost all the DEGs involved in energy consumption pathways, such as the cell cycle, nucleic acid processes, and mitosis and meiosis, were down-regulated in the mutant (Fig. 5E–G, Supplementary Figs S10C, S11A, B).

As BY1 catalyses the formation of DAHP from PEP and E4-P and participates in the first step of the shikimate pathway, we investigated the expression patterns of genes involved in this pathway, together with those involved in the pentose phosphate pathway and glycolysis, and found that all were significantly up-regulated in *by1*compared the WT (Supplementary Fig. S12A–C). In addition, we examined the expression patterns of genes downstream in the general phenylpropanoid pathway and found that that they were also up-regulated in the mutant (Supplementary Fig. S12D). Finally, to validate the reliability of the transcriptome data, we randomly selected 10 up-regulated and 10 down-regulated DEGs and carried out RT-qPCR analysis. As expected, the relative expression levels were generally consistent with the mRNA-seq data (Supplementary Fig. S11C, D), suggesting that the data were of high quality.

**Fig. 6.**
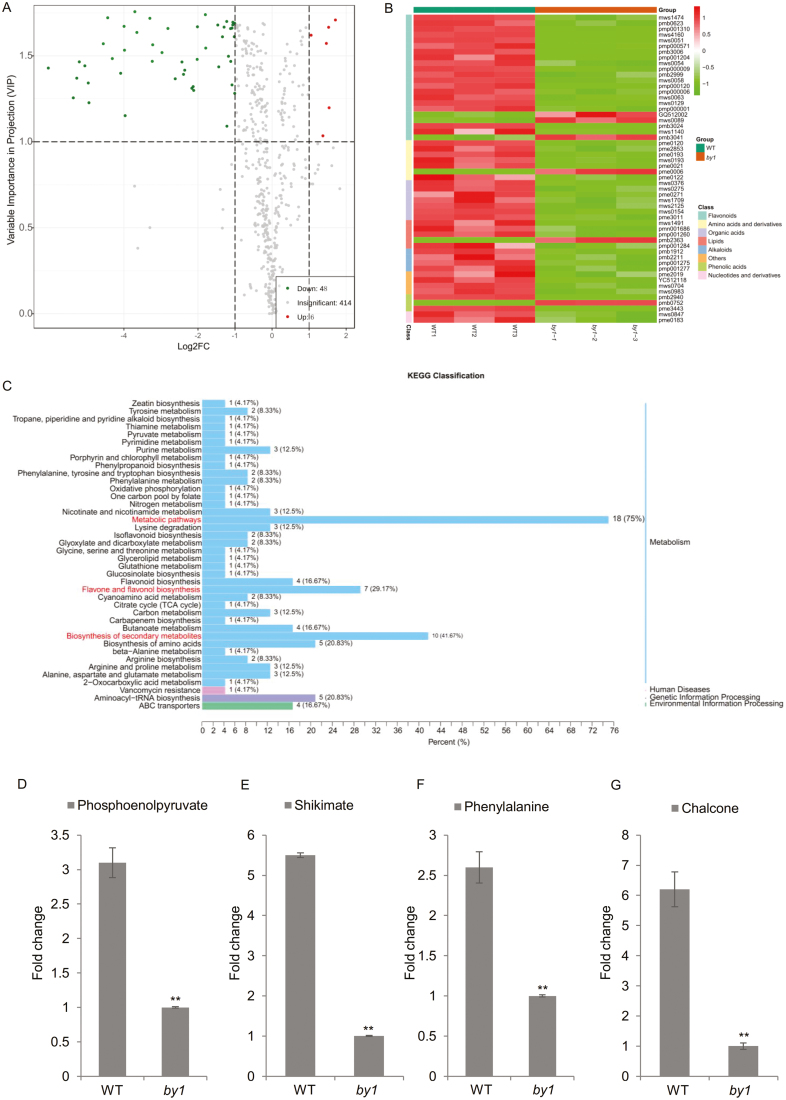
Metabolic profiling analysis of sorghum wild-type *BY1* and the *by1* mutant. (A) Volcano plot of the different metabolites. For each metabolite, the variable importance in projection (VIP) value is plotted against the logarithm of the quantitative difference multiple of the metabolite in two samples: the larger the absolute value, the greater the difference multiple of the content of the metabolite. The larger the VIP value, the more significant the difference in the metabolite levels. Metabolites down-regulated in *by1* relative to the wild-type (WT) are shown in green, metabolites up-regulated are shown in red, and those with no significant difference are shown in gray. (B) Clustering heatmap of metabolites with significantly different contents (MSDCs) in *by1* compared with the WT. The metabolite classes are grouped according to the color scale and their contents are indicated by the red–green scale. WT lines are to the left and *by1* lines are to the right. (C) KEGG classification of MSDCs. The number of the MSDCs annotated to each pathway is shown together with its percentage of the total number of MSDCs. (D–G) Fold-changes of (D) phosphoenolpyruvate, (E) shikimate, (F) phenylalanine, and (G) chalcone levels between the WT and *by1*. Data are means (±SD), *n*=3. Significant differences were determined using two-tailed Student’s *t*-test: **P<0.01.

To further assess the influence of *BY1* on metabolites in sorghum, we performed a widely targeted metabolomics analysis using a UPLC-MS/MS detection platform. We detected 414 differential metabolites in leaves of the WT compared with *by1*, (Fig. 6A) including flavonoids, organic acids, amino acids and their derivatives, phenolic acids, lipids, alkaloids, and nucleotides and their derivatives. Principal component analysis (PCA) revealed an obvious separation between the WT and *by1* samples, indicating that they differed significantly (Supplementary Fig. S13A).

Based on the criteria of a fold-change of ≥2 or ≤0.5, we identified 54 metabolites with significantly different levels between the genotypes, with six having higher levels and 48 having lower levels in *by1* (Fig. 6A; Supplementary Table S2); we termed these 54 metabolites as ‘metabolites with significantly different contents’ (MSDCs). The MSDCs were clustered into six subclasses based on the changes in their levels (Supplementary Fig. S13B). To observe these changes more clearly, we normalized the levels, divided them into different classes, and drew a heatmap using the R software. The levels of most MSDCs were significantly reduced in *by1* compared with the WT. The largest class was flavonoids (Fig. 6B). KEGG analysis revealed that the MSDCs were enriched in various pathways, with the most highly enriched being the metabolic pathways in the biosynthesis of secondary metabolites, and in flavone and flavonol biosynthesis (Fig. 6C, Supplementary Fig. S13C).

Since BY1 is a functional enzyme that participates in the shikimate pathway, which functions downstream of the pentose phosphate pathway and glycolysis, we investigated the levels of metabolites in these primary metabolic pathways. Compared to the WT, the content of shikimic acid (the product of the shikimate pathway) was reduced by ~5.5-fold in *by1*, and the content of PEP (the intermediate product of glycolysis) was reduced by ~3.1-fold (Fig. 6D, E). In addition, the levels of many metabolites in pathways downstream of the shikimate pathway were also significantly reduced in *by1*. For example, the content of phenylalanine in the AAA pathway was ~2.6-fold lower and the content of chalcone in the phenylpropane pathway was ~6.2-fold lower (Fig. 6F, G). Finally, most of the MSDCs were secondary metabolites involved in phenylpropane metabolism, which also occurs downstream of the shikimate pathway. Of these metabolites, all four alkaloids and 19 of the 22 flavonoids accumulated to lower levels in *by1* than in the WT (Supplementary Table S2). These results indicated that the mutation in *by1* significantly influenced the homeostasis of primary and secondary metabolites in sorghum.

## Discussion

Sorghum is one of the world’s most important crops, providing staple food for more than 500 million people, as well as serving as a model energy plant with high biological yield (Anami *et al.*, 2015). Biomass and grain yield are thus key agronomic traits in sorghum; however, the molecular mechanisms that regulate them are not well understood. Here, we isolated the *by1* mutant, cloned *BY1*, and examined its function. *BY1*, a constitutively expressed gene, encodes a 3-deoxy-D-*arabino*-heptulosonate-7-phosphate synthase (DAHPS) that is localized to chloroplasts. Transcriptome analysis and metabolic profiling shed light on how BY1 regulates sorghum biomass and grain yield, and provided important insights into the relationship between plant development and metabolism.

### Abnormal cell cycle and floral organ development are responsible for the phenotype of the *by1* mutant

The biomass of *by1* was reduced compared to the wild-type (WT) for several reasons, including morphological abnormalities, reduced plant height, thin stems, and narrow leaves (Fig. 1A, C). In addition, poorer development of the top of the panicle, shorter panicle length, and lower seed-setting rate in *by1* led to decreased grain yield per plant (Fig. 1B, D, Supplementary Fig. S3). At the microscopic level, paraffin-sectioning and SEM analysis revealed that the decreased plant height, thinner stems, and narrower leaves in *by1* were due to inhibited cell elongation (Fig. 2A–I, Supplementary Figs S1A–D, S2), which ultimately resulted in the reduced biomass. The decreased grain yield in *by1* was due to poor anther development, incomplete pollen development, and decreased pollen quality and quantity (Fig. 2J–Q). At the gene-expression level, transcriptome analysis indicated the presence of many DEGs related to the cell cycle, pollen development, nucleic acids, and mitosis and meiosis, most of which were down-regulated in *by1* relative to the WT (Fig. 5A, B, E, Supplementary Figs S9D, S10C, S11A, B); these findings were all consistent with the phenotypes of the mutant.

### A single-nucleotide mutation leads to BY1 loss of function

We cloned sorghum *BY1* using map-based cloning and based on Pfam analysis, determined that it encodes a member of the DAHP_synth_2_supperfamily (Fig. 3A–E). Homology analysis indicated that the DAHP_synth_2_supperfamily is widespread in higher plants (Supplementary Fig. S6), whilst motif analysis and multiple sequence alignment demonstrated that the amino acid mutation site in *by1* is located in a region that is highly conserved among higher plants (Fig. 3F). Comparison of 164 sorghum germplasm strains and 20 randomly selected recessive plants from an F_2_ population suggested that the mutation in *by1* may not exist in natural sorghum accessions, and that the mutation site was likely to play an important role in maintaining the normal function of *BY1* (Supplementary Data S1, Supplementary Fig. S5).

Analyses of gene expression and subcellular localization indicated that *BY1* was constitutively expressed and that BY1 was specifically localized to chloroplasts, with its subcellular location not being affected by the *by1* mutation (Fig. 4A, B). The conversion of the 192nd amino acid from Pro to Leu strongly altered the tertiary structure of the BY1 protein (Fig. 4C, Supplementary Fig. S15), which abolished its enzymatic activity (Fig. 4D, E). Finally, the phenotypes of all the independently gene-edited rice plants that we examined were highly similar to those of the sorghum *by1* mutant (Fig. 3G–I, Supplementary Fig. S7). Taken together, results demonstrated that Sobic.002G379600 was *BY1*, that this gene influenced biomass and grain yield in sorghum, and that a single-nucleotide mutation was responsible for its loss of function.

### BY1 participates in the shikimate pathway and influences primary and secondary metabolism

The shikimate pathway only exists in microorganisms and plants (Bickel and Schultz, 1979; Maeda and Dudareva, 2012). This pathway initiates from PEP and E-4-P and ends with chorismate (CHR), and it involves seven reactions catalysed by six different enzymes (Supplementary Fig. S14A). Phenylalanine and tryptophan are essential amino acids for animals that must be obtained from food, and tyrosine is synthesized from phenylalanine. In plants, the shikimate pathway is a more widespread metabolic pathway than nitrogen fixation and photosynthesis (Jensen, 2006; Maeda and Dudareva, 2012), as it is required to generate aromatic amino acids (AAAs), which are crucial for both plants and animals. The shikimate pathway plays an important role in regulating primary and secondary metabolism (Zabalza *et al.*, 2017).

Our results provide several lines of evidence that BY1 is involved in the shikimate pathway and influences primary and secondary metabolism (Supplementary Fig. S14). First, we have demonstrated that *BY1* encodes a DAHPS protein, and that the mutation in *by1* leads to loss of function (Figs 3, 4C, D) and the induction of genes involved in the shikimate pathway (Supplementary Figs S12C, S14B). Second, transcriptome analysis indicated that most genes related to photosynthesis, glycolysis, and the pentose phosphate pathway upstream of the shikimate pathway, and the phenylpropane metabolism pathway downstream of the shikimate pathway, were significantly up-regulated in the *by1* mutant (Fig. 5C, D, Supplementary Figs S10A, B, S12A, B, D). Third, metabolic profiling revealed that the contents of several primary metabolites were reduced in *by1* compared with the WT. For example, levels of shikimate, PEP, and phenylalanine in the mutant were reduced by ~5.5-fold, ~3.1-fold, and ~2.6-fold, respectively (Fig. 6D–F, Supplementary Fig. S14A), and the levels of most flavonoids (secondary metabolites) were also significantly reduced in *by1* (Fig. 6B–G, Supplementary Fig. S14A, Supplementary Table S2).

Based on our comprehensive analyses of the changes in metabolite contents in the shikimate pathway and its upstream and downstream pathways, together with the changes in the expression of genes related to these pathways, we propose a metabolic map that highlights the important regulatory role played by BY1 (Fig. S14). , which

In summary, our results indicated that BY1 participates in the shikimate pathway and regulates the flow of primary carbon metabolites into the biosynthesis of CHR, which then generates AAAs and secondary metabolites.

### A possible feedback regulation mechanism in sorghum primary and secondary metabolism

The results of transcriptome analysis and metabolic profiling indicated that genes involved in glycolysis, and the pentose phosphate, shikimate and phenylpropanoid pathways were up-regulated in the *by1* mutant. However, the levels of metabolites in these pathways were reduced (PEP, shikimate, chalcone, and flavonoids). These results suggest that a feedback mechanism might exist in sorghum to regulate primary and secondary metabolism. In the WT, the functioning of BY1 was required for maintenance of the homeostasis in gene expression and the flux of metabolites, and hence for normal growth and development. However, in the *by1* mutant, the abnormal functioning of BY1 and the compromised shikimate pathway may have triggered the metabolic adjustments that were observed in primary and secondary metabolism, resulting in the decreased contents of the associated metabolites. The decreased metabolic levels, or changes in associated redox states, may have been sensed by as yet unknown mechanisms to regulate gene expression in order to restore the carbon flux through the pathways. Our results agree with previous findings that reduced levels of AAAs or downstream products induce gene expression in the shikimate pathway (Maeda and Dudareva, 2012).

### A model for the role of BY1 in regulating plant growth and development in sorghum

More than 90% of the total metabolic energy in bacteria is used to synthesize proteins (Herrmann and Weaver, 1999), and the shikimate pathway is used almost exclusively to synthesize AAAs, which are further used to synthesize proteins (Garbe *et al.*,1991; Herrmann and Weaver, 1999).In contrast, in higher plants most AAAs synthesized by the shikimate pathway are used as precursors of secondary metabolites (Maeda and Dudareva, 2012). Approximately 20% of the carbon fixed in green plants is estimated to pass through the shikimate pathway. Every year, ~7×10^15^ kg of carbon enters to this pathway worldwide, most of which eventually forms AAAs, flavonoids, vitamins, lignin, phenols, and other secondary metabolites that regulate plant growth and development (Rippert *et al.*, 2004).

The amount and types of secondary metabolites synthesized by the shikimate pathway vary greatly from species to species (Campbell *et al.*, 2004). Flavonoids are secondary metabolites that are widely distributed in plants. In addition to functioning as antioxidants that help plants cope with abiotic stress, they regulate plant growth and development by participating in the transmission of hormonal signals, promoting pollen tube germination, and sensing microbial signals (Taylor and Grotewold, 2005; Kuhn *et al.*, 2011; Brunetti *et al.*, 2013).

**Fig. 7.**
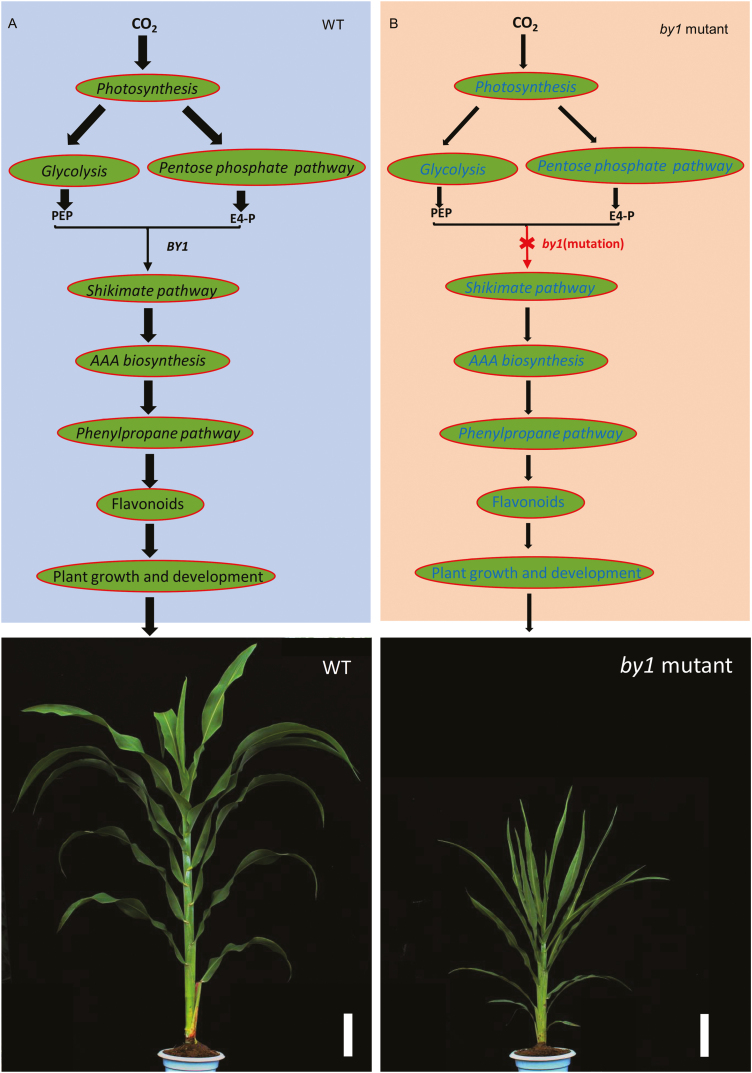
A working model of the role of BY1 in regulating sorghum growth and development. (A) In the wild-type (WT), a portion of carbon dioxide fixed during photosynthesis is transformed into phosphoenolpyruvate (PEP) and erythritol-4-phosphate (E4-P) through glycolysis and the pentose phosphate pathway, respectively. PEP and E4-P enter the shikimate pathway under the control of BY1, pass through aromatic amino acid (AAA) biosynthesis and the flavonoid metabolism pathway, and form flavonoids, which regulate plant growth and development. (B) In the *BY1* loss-of-function mutant (*by1*), the shikimate pathway is weakened, and feedback regulation leads to a decrease in the flow of carbon source the shikimate pathway, which leads to abnormal contents of secondary metabolites, thereby adversely affecting plant growth and development. The widths of the black arrows represent the amount of the carbon flow in the pathway. Scale bars in the images are 10 cm.

Based on the findings of our study, we propose a model to describe how BY1 regulates growth and development in sorghum (Fig. 7). According to this model, a portion of the carbon fixed by photosynthesis flows into the shikimate pathway through glycolysis and the pentose phosphate pathway and is used to form AAAs. These AAAs are then used to synthesize various secondary metabolites (especially flavonoids) via phenylpropane metabolism to regulate plant growth and development (Fig. 7A). In the *BY1* loss-of-function mutant, the shikimate pathway is affected, leading to feedback regulation of the upstream pathways including photosynthesis, glycolysis, and the pentose phosphate pathway. This process decreases the flow of carbon sources into the shikimate pathway, resulting in decreased levels of secondary metabolites such as flavonoids, which ultimately affects plant growth and development (Fig. 7B). This model shows the regulation mechanism of BY1 on the biomass and grain yield of sorghum, highlighting its potential use for the future molecular design and breeding of sorghum, and it provides important insights into the relationship between plant development and metabolism.

## Supplementary data

Supplementary data are available at *JXB* online.

Fig. S1. Morphology of panicle neck internodes, grains, and roots of the wild-type and *by1* mutant.

Fig. S2. Morphology leaf veins of the wild-type and *by1* mutant.

Fig. S3. Phenotypes of young panicles in the wild-type and *by1* mutant before flowering.

Fig. S4. Phenotypes of fertile florets in the wild-type and *by1* mutant.

Fig. S5. Sequencing and alignment of the mutation site among recessive individual plants, the *by1* mutant, and the wild-type.

Fig. S6. Phylogenetic analysis of BY1 and its homologs.

Fig. S7. Phenotypic comparison between wild-type rice ZH11 and plants genome-edited plants expressing *by1*.

Fig. S8. Sequencing of *BY1*.

Fig. S9. Transcriptome analysis of *BY1*.

Fig. S10. Details of DEGs related to photosynthesis, phenylpropane metabolism, and the cell cycle.

Fig. S11. Details of DEGs related to nucleic acids, and mitosis and meiosis, together with RT-qPCR verification.

Fig. S12. RPKM values for genes related to glycolysis, and the pentose phosphate, shikimate, and phenylpropanoid pathways.

Fig. S13. Metabolic profiling of *BY1*.

Fig. S14. Metabolic map of pathways related to *BY1*.

Fig. S15. Comparison of the tertiary structures of BY1 in the wild-type and the *by1* mutant.

Table S1. Comparison of agronomic traits between the wild-type and the *by1* mutant.

Table S2. Characterization of metabolites present at different levels in the wild-type and the *by1* mutant.

Table S3. Primers used in this study.

Data S1. List of sorghum varieties used to verify the mutation site in *by1*.

Data S2. Details of the 1785 DEGs identified between wild-type and the *by1* mutant.

eraa275_suppl_Supplementary_File001

eraa275_suppl_Supplementary_Data-S1

eraa275_suppl_Supplementary_Data-S2

eraa275_suppl_Supplementary_Table-S1-S3

## References

[CIT0001] Aharoni A, Galili G 2011 Metabolic engineering of the plant primary–secondary metabolism interface. Current Opinion in Biotechnology 22, 239–244. 21144730 10.1016/j.copbio.2010.11.004

[CIT0002] Anami SE, Zhang LM, Xia Y, Zhang YM, Liu ZQ, Jing HC 2015 Sweet sorghum ideotypes: genetic improvement of the biofuel syndrome. Food and Energy Security 4, 159–177.

[CIT0003] Arcuri HA, Canduri F, Pereira JH, da Silveira NJ, Camera Júnior JC, de Oliveira JS, Basso LA, Palma MS, Santos DS, de Azevedo Júnior WF 2004 Molecular models for shikimate pathway enzymes of *Xylella fastidiosa*. Biochemical and Biophysical Research Communications 320, 979–991. 15240145 10.1016/j.bbrc.2004.05.220

[CIT0004] Bentley R, Haslam E 1990 The shikimate pathway — a metabolic tree with many branches. Critical Reviews in Biochemistry 25, 307–384. 10.3109/10409239009090615 2279393

[CIT0005] Bickel H, Schultz G 1979 Shikimate pathway regulation in suspensions of intact spinach chloroplasts. Phytochemistry 18, 498–499.

[CIT0006] Brunetti C, Di Ferdinando M, Fini A, Pollastri S, Tattini M 2013 Flavonoids as antioxidants and developmental regulators: relative significance in plants and humans. International Journal of Molecular Sciences 14, 3540–3555. 23434657 10.3390/ijms14023540 PMC3588057

[CIT0007] Campbell SA, Richards TA, Mui EJ, Samuel BU, Coggins JR, McLeod R, Roberts CW 2004 A complete shikimate pathway in *Toxoplasma gondii*: an ancient eukaryotic innovation. International Journal for Parasitology 34, 5–13. 14711585 10.1016/j.ijpara.2003.10.006

[CIT0008] Chávez-Béjar MI, Lara AR, López H, Hernández-Chávez G, Martinez A, Ramírez OT, Bolívar F, Gosset G 2008 Metabolic engineering of *Escherichia coli* for L-tyrosine production by expression of genes coding for the chorismate mutase domain of the native chorismate mutase-prephenate dehydratase and a cyclohexadienyl dehydrogenase from *Zymomonas mobilis*. Applied and Environmental Microbiology 74, 3284–3290. 18344329 10.1128/AEM.02456-07 PMC2394925

[CIT0009] Chen W, Gong L, Guo Z, Wang W, Zhang H, Liu X, Yu S, Xiong L, Luo J 2013 A novel integrated method for large-scale detection, identification, and quantification of widely targeted metabolites: application in the study of rice metabolomics. Molecular Plant 6, 1769–1780. 23702596 10.1093/mp/sst080

[CIT0010] Cifuentes R, Bressani R, Rolz C 2014 The potential of sweet sorghum as a source of ethanol and protein. Energy for Sustainable Development 21, 13–19.

[CIT0011] Crooks GE, Hon G, Chandonia JM, Brenner SE 2004 WebLogo: a sequence logo generator. Genome Research 14, 1188–1190. 15173120 10.1101/gr.849004 PMC419797

[CIT0012] Garbe T, Servos S, Hawkins A, Dimitriadis G, Young D, Dougan G, Charles I 1991 The *Mycobacterium tuberculosis* shikimate pathway genes: evolutionary relationship between biosynthetic and catabolic 3-dehydroquinases. Molecular & General Genetics 228, 385–392. 1910148 10.1007/BF00260631

[CIT0013] Gilbert N 2009 Averting a climate-led food crisis in Africa. Nature doi:10.1038/news.2009.585.

[CIT0014] Hay ME 1996 Marine chemical ecology: what’s known and what’s next? Journal of Experimental Marine Biology and Ecology 200, 103–134.

[CIT0015] Herrmann KM 1995 The shikimate pathway pathway: early steps in the biosynthesis of aromatic compounds. The Plant Cell 7, 907–919. 12242393 10.1105/tpc.7.7.907 PMC160886

[CIT0016] Herrmann KM, Weaver LM 1999 The shikimate pathway. Annual Review of Plant Physiology and Plant Molecular Biology 50, 473–503. 10.1146/annurev.arplant.50.1.473 15012217

[CIT0017] Jensen RA 2006 The shikimate/arogenate pathway: link between carbohydrate metabolism and secondary metabolism. Physiologia Plantarum 66, 164–168.

[CIT0018] Keith B, Dong XN, Ausubel FM, Fink GR 1991 Differential induction of 3-deoxy-d-*arabino*-heptulosonate 7-phosphate synthase genes in *Arabidopsis thaliana* by wounding and pathogenic attack. Proceedings of the National Academy of Sciences, USA 88, 8821–8825. 10.1073/pnas.88.19.8821 PMC52602 1681544

[CIT0019] Kuhn BM, Geisler M, Bigler L, Ringli C 2011 Flavonols accumulate asymmetrically and affect auxin transport in *Arabidopsis*. Plant Physiology 156, 585–595. 21502189 10.1104/pp.111.175976 PMC3177260

[CIT0020] Lawrence CJ, Walbot V 2007 Translational genomics for bioenergy production from fuelstock grasses: maize as the model species. The Plant Cell 19, 2091–2094. 17660357 10.1105/tpc.107.053660 PMC1955697

[CIT0021] Li ML, Yuyama N, Luo L, Hirata M, Cai H 2009 *In silico* mapping of 1758 new SSR markers developed from public genomic sequences for sorghum. Molecular Breeding 24, 41–47.

[CIT0022] Light SH, Anderson WF 2013 The diversity of allosteric controls at the gateway to aromatic amino acid biosynthesis. Protein Science 22, 395–404. 23400945 10.1002/pro.2233 PMC3610045

[CIT0023] Liu Q, Liu H, Gong Y, et al 2017 An atypical thioredoxin imparts early resistance to sugarcane mosaic virus in maize. Molecular Plant 10, 483–497. 28216424 10.1016/j.molp.2017.02.002

[CIT0024] Livak KJ, Schmittgen TD 2001 Analysis of relative gene expression data using real-time quantitative PCR and the 2^–∆∆*C*T^ method. Methods 25, 402–408. 11846609 10.1006/meth.2001.1262

[CIT0025] Ma N, Wei L, Fan Y, Hua Q 2012 Heterologous expression and characterization of soluble recombinant 3-deoxy-D-arabino-heptulosonate-7-phosphate synthase from *Actinosynnema pretiosum* ssp. *auranticum* ATCC31565 through co-expression with Chaperones in *Escherichia coli*. Protein Expression and Purification 82, 263–269. 22326798 10.1016/j.pep.2012.01.013

[CIT0026] Maeda H, Dudareva N 2012 The shikimate pathway and aromatic amino acid biosynthesis in plants. Annual Review of Plant Biology 63, 73–105. 10.1146/annurev-arplant-042811-105439 22554242

[CIT0027] Maeda H, Yoo H, Dudareva N 2011 Prephenate aminotransferase directs plant phenylalanine biosynthesis via arogenate. Nature Chemical Biology 7, 19–21. 21102469 10.1038/nchembio.485

[CIT0028] Michelmore RW, Paran I, Kesseli RV 1991 Identification of markers linked to disease-resistance genes by bulked segregant analysis: a rapid method to detect markers in specific genomic regions by using segregating populations. Proceedings of the National Academy of Sciences, USA 88, 9828–9832. 10.1073/pnas.88.21.9828 PMC52814 1682921

[CIT0029] Murray MG, Thompson WF 1980 Rapid isolation of high molecular weight plant DNA. Nucleic Acids Research 8, 4321–4325. 7433111 10.1093/nar/8.19.4321 PMC324241

[CIT0030] Nazmi AR, Schofield LR, Dobson RC, Jameson GB, Parker EJ 2014 Destabilization of the homotetrameric assembly of 3-deoxy-D-*arabino*-heptulosonate-7-phosphate synthase from the hyperthermophile *Pyrococcus furiosus* enhances enzymatic activity. Journal of Molecular Biology 426, 656–673. 24239948 10.1016/j.jmb.2013.11.008

[CIT0031] Pagor R, Sikdar MSL, Moon ES, Kim JS 2015 Characterization of a gene encoding 3-deoxy-D-*arabino*-heptulosonate-phosphate synthase from rice. Journal of Biological Sciences 4, 461–466.

[CIT0032] Palakolanu SR, Dumbala SR, Kaliamoorthy S, Pooja BM, Vincent V, Sharma KK 2016 Evaluation of sorghum [*Sorghum bicolor* (L.)] reference genes in various tissues and under abiotic stress conditions for quantitative real-time PCR data normalization. Frontiers in Plant Science 7, 529. 27200008 10.3389/fpls.2016.00529 PMC4843019

[CIT0033] Paterson AH, Bowers JE, Bruggmann R, et al 2009 The *Sorghum bicolor* genome and the diversification of grasses. Nature 457, 551–556. 19189423 10.1038/nature07723

[CIT0034] Rippert P, Scimemi C, Dubald M, Matringe M 2004 Engineering plant shikimate pathway for production of tocotrienol and improving herbicide resistance. Plant Physiology 134, 92–100. 14684842 10.1104/pp.103.032441 PMC316290

[CIT0035] Roberts F, Roberts CW, Johnson JJ, et al 1998 Evidence for the shikimate pathway in apicomplexan parasites. Nature 393, 801–805. 9655396 10.1038/31723

[CIT0036] Takaki M, Tan L, Murakami T, Tang YQ, Sun ZY, Morimura S, Kida K, Kida K 2015 Production of biofuels from sweet sorghum juice via ethanol–methane two-stage fermentation. Industrial Crops and Products 63, 329–336.

[CIT0037] Tamura K, Peterson D, Peterson N, Stecher G, Nei M, Kumar S 2011 MEGA5: molecular evolutionary genetics analysis using maximum likelihood, evolutionary distance, and maximum parsimony methods. Molecular Biology and Evolution 28, 2731–2739. 21546353 10.1093/molbev/msr121 PMC3203626

[CIT0038] Taylor LP, Grotewold E 2005 Flavonoids as developmental regulators. Current Opinion in Plant Biology 8, 317–323. 15860429 10.1016/j.pbi.2005.03.005

[CIT0039] Thompson JD, Higgins DG, Gibson TJ 1994 CLUSTAL W: improving the sensitivity of progressive multiple sequence alignment through sequence weighting, position-specific gap penalties and weight matrix choice. Nucleic Acids Research 22, 4673–4680. 7984417 10.1093/nar/22.22.4673 PMC308517

[CIT0040] Trapnell C, Williams BA, Pertea G, Mortazavi A, Kwan G, van Baren MJ, Salzberg SL, Wold BJ, Pachter L 2010 Transcript assembly and quantification by RNA-seq reveals unannotated transcripts and isoform switching during cell differentiation. Nature Biotechnology 28, 511–515. 10.1038/nbt.1621 PMC3146043 20436464

[CIT0041] Tuskan GA, Difazio S, Jansson S, et al 2006 The genome of black cottonwood, *Populus trichocarpa* (Torr. & Gray). Science 313, 1596–1604. 16973872 10.1126/science.1128691

[CIT0042] Tzin V, Galili G 2010 New insights into the shikimate and aromatic amino acids biosynthesis pathways in plants. Molecular Plant 3, 956–972. 20817774 10.1093/mp/ssq048

[CIT0043] Tzin V, Malitsky S, Aharoni A, Galili G 2009 Expression of a bacterial bi-functional chorismate mutase/prephenate dehydratase modulates primary and secondary metabolism associated with aromatic amino acids in *Arabidopsis*. The Plant Journal 60, 156–167. 19508381 10.1111/j.1365-313X.2009.03945.x

[CIT0044] Tzin V, Malitsky S, Ben Zvi MM, Bedair M, Sumner L, Aharoni A, Galili G 2012 Expression of a bacterial feedback-insensitive 3-deoxy-D-*arabino*-heptulosonate 7-phosphate synthase of the shikimate pathway in Arabidopsis elucidates potential metabolic bottlenecks between primary and secondary metabolism. New Phytologist 194, 430–439. 22296303 10.1111/j.1469-8137.2012.04052.x

[CIT0045] Wang Y, Herrmann KM, Weller SC, Goldsbrough PB 1991 Cloning and nucleotide sequence of a complementary DNA encoding 3-deoxy-D-*arabino*-heptulosonate 7-phosphate synthase from tobacco. Plant Physiology 97, 847–848. 16668482 10.1104/pp.97.2.847 PMC1081090

[CIT0046] Xie Q, Xu Z 2019 Sustainable agriculture: from sweet sorghum planting and ensiling to ruminant feeding. Molecular Plant 12, 603–606. 31002980 10.1016/j.molp.2019.04.001

[CIT0047] Yamada T, Matsuda F, Kasai K, Fukuoka S, Kitamura K, Tozawa Y, Miyagawa H, Wakasa K 2008 Mutation of a rice gene encoding a phenylalanine biosynthetic enzyme results in accumulation of phenylalanine and tryptophan. The Plant Cell 20, 1316–1329. 18487352 10.1105/tpc.107.057455 PMC2438470

[CIT0048] Yonemaru J, Ando T, Mizubayashi T, Kasuga S, Matsumoto T, Yano M 2009 Development of genome-wide simple sequence repeat markers using whole-genome shotgun sequences of sorghum (*Sorghum bicolor* (L.) Moench). DNA Research 16, 187–193. 19363056 10.1093/dnares/dsp005 PMC2695772

[CIT0049] Zabalza A, Orcaray L, Fernández-Escalada M, Zulet-González A, Royuela M 2017 The pattern of shikimate pathway and phenylpropanoids after inhibition by glyphosate or quinate feeding in pea roots. Pesticide Biochemistry and Physiology 141, 96–102. 28911748 10.1016/j.pestbp.2016.12.005

[CIT0050] Zhang WN, He JJ, Pan QH, Han FL, Duan CQ 2011 Separation and character analysis of anthocyanins from mulberry (*Morus alba* L.) pomace. Czech Journal of Food Sciences 29, 268–276.

[CIT0051] Zhao J, Herrmann KM 1992 Cloning and sequencing of a second cDNA encoding 3-deoxy-D-*arabino*-heptulosonate 7-phosphate synthase from *Solanum tuberosum* L. Plant Physiology 100, 1075–1076. 16653023 10.1104/pp.100.2.1075 PMC1075672

[CIT0052] Zhu G, Wang S, Huang Z, et al 2018 Rewiring of the fruit metabolome in tomato breeding. Cell 172, 249–261.e12. 29328914 10.1016/j.cell.2017.12.019

