# Supplementary material for: BIOMASS YIELD 1 regulates sorghum biomass and grain yield via the shikimate pathway
Source: J Exp Bot. 2020 Jun 4;71(18):5506–20. doi: 10.1093/jxb/eraa275 (PMC7501818; doi:10.1093/jxb/eraa275)
Supplement: eraa275_suppl_Supplementary_File001 [file eraa275_suppl_supplementary_file001.pdf]

Supplementary Information

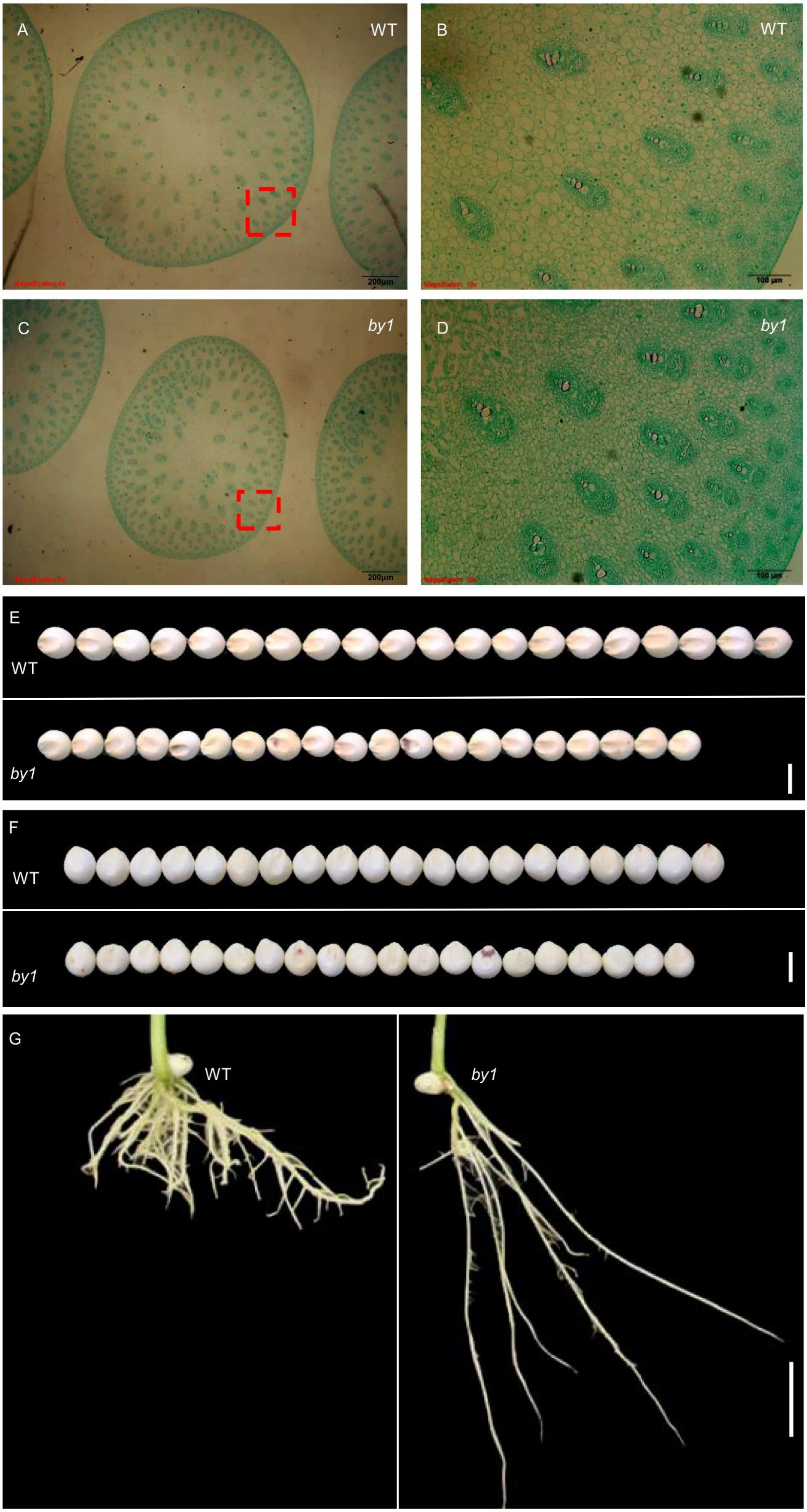

Supplemental Figure S1 Cross section of the panicle neck internodes, grain length, grain width and root phenotypes comparison between WT and *by1* mutant.

(A) Cross-sections of panicle neck internodes in wild type (WT). Bar=200 μm.

(B) Enlarged view of the red dotted box part in A. Bar=100 μm.

(C) Cross-sections of panicle neck internodes in *by1* mutant. Bar=200 μm.

(D) Enlarged view of the red dotted box part in C. Bar=100 μm.

(E) Grain length comparison between WT and *by1* mutant. Bar=3 mm.

(F) Grain width comparison between WT and *by1* mutant. Bar=3 mm.

(G) Root phenotypes comparison between WT and *by1* mutant. Bar=1 cm.

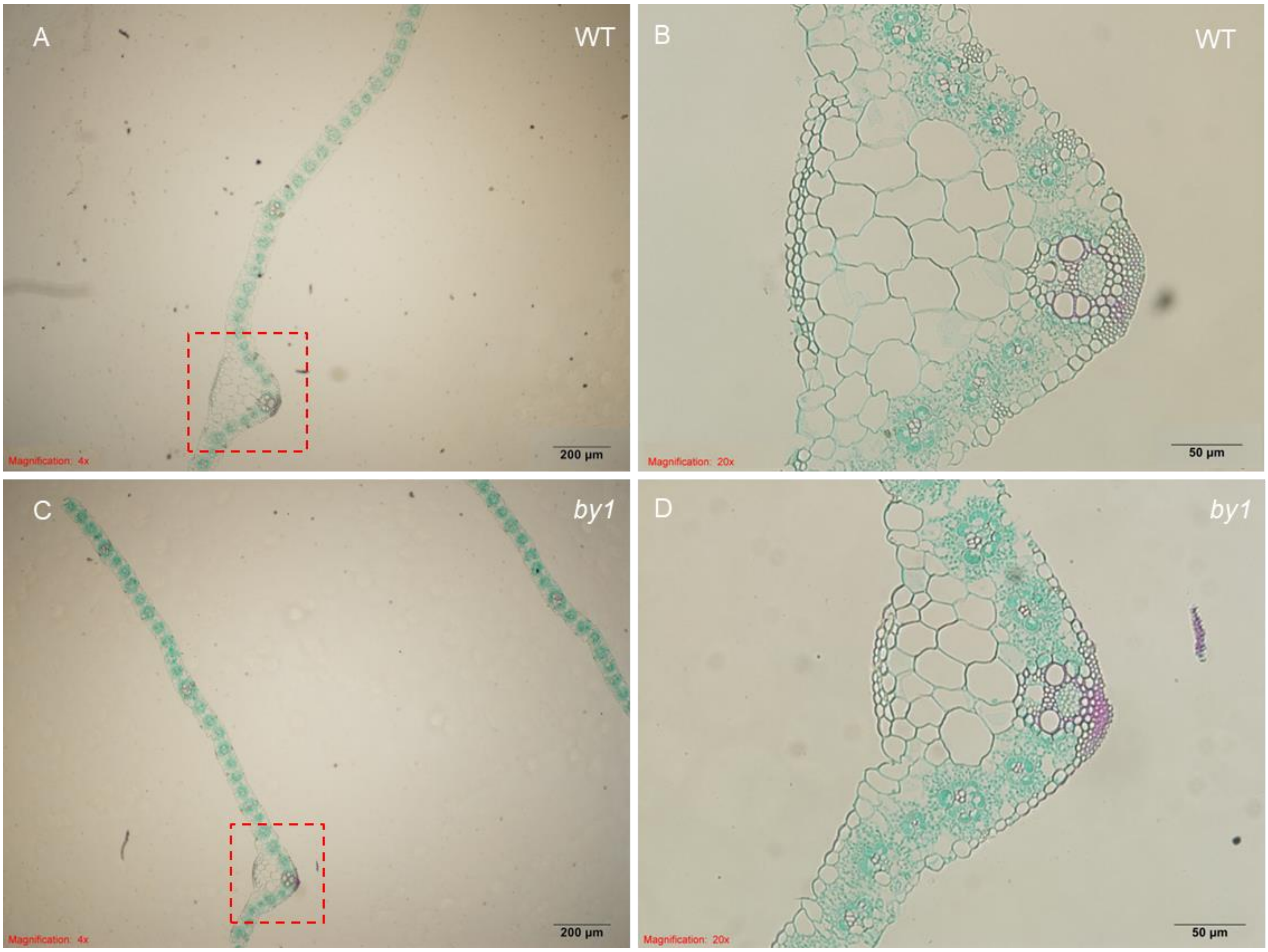

Supplemental Figure S2 Cross-section comparison of veins between WT and *by1* mutant.

(A) Cross-sections of veins in wide type (WT). Red dotted box indicates main vein. Bar=200 μm.

(B) Enlarged view of the red dotted box part in A. Bar=50 μm.

(C) Cross-sections of veins in *by1* mutant. Red dotted box indicates main vein. Bar=200 μm.

(D) Enlarged view of the red dotted box part in C. Bar=50 μm.

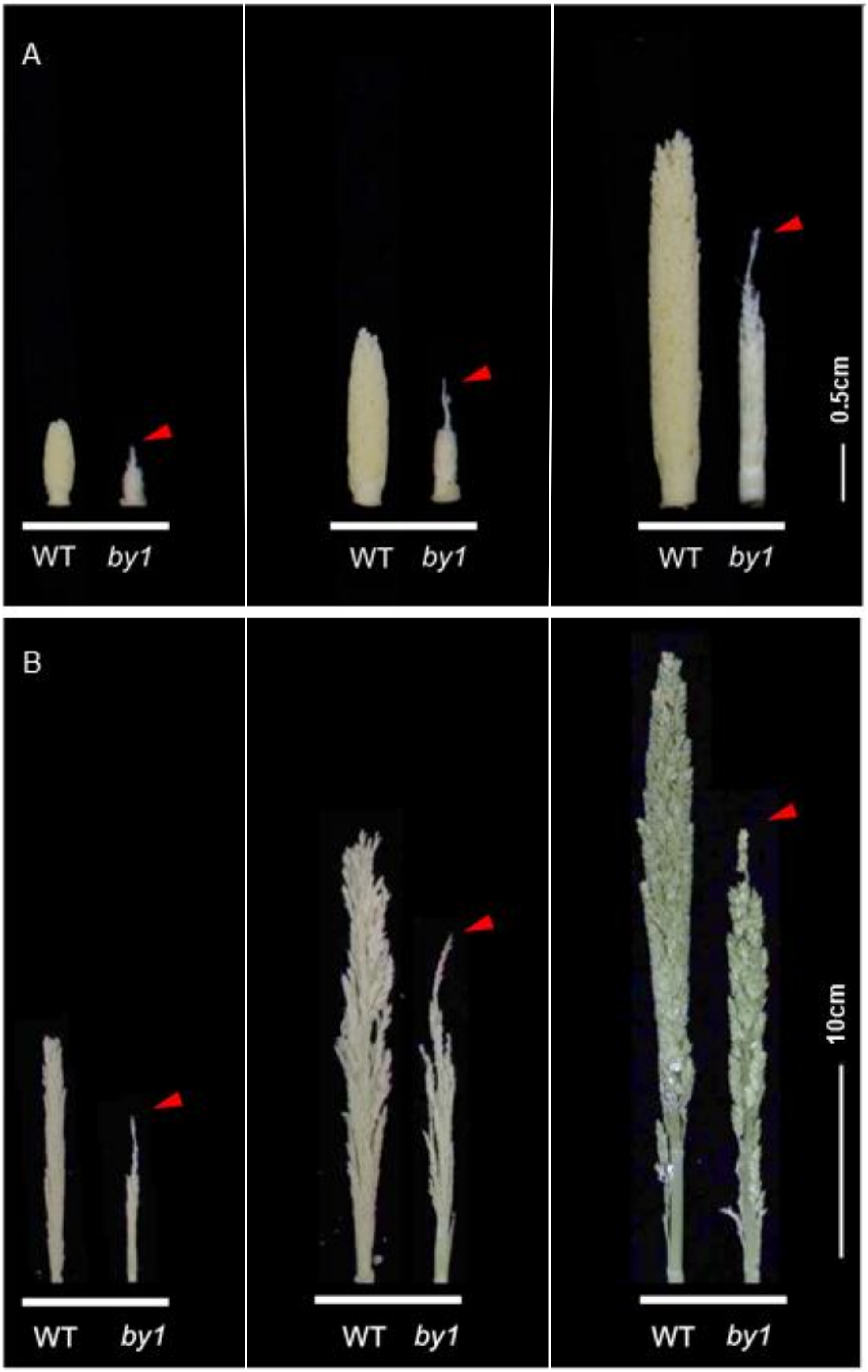

Supplemental Figure S3 Young-panicle phenotypes comparison between WT and *by1* mutant before flowering.

(A) Young-panicle phenotypes comparison between WT and *by1* mutant at the early booting stage. Bar=0.5 cm. The red arrowheads indicate the top of the abnormal development of *by1* panicles.

(B) Young-panicle phenotypes comparison between WT and *by1* mutant at the middle and later booting stages. Bar=10 cm. The red arrowheads indicate the top of the abnormal development of *by1* panicles.

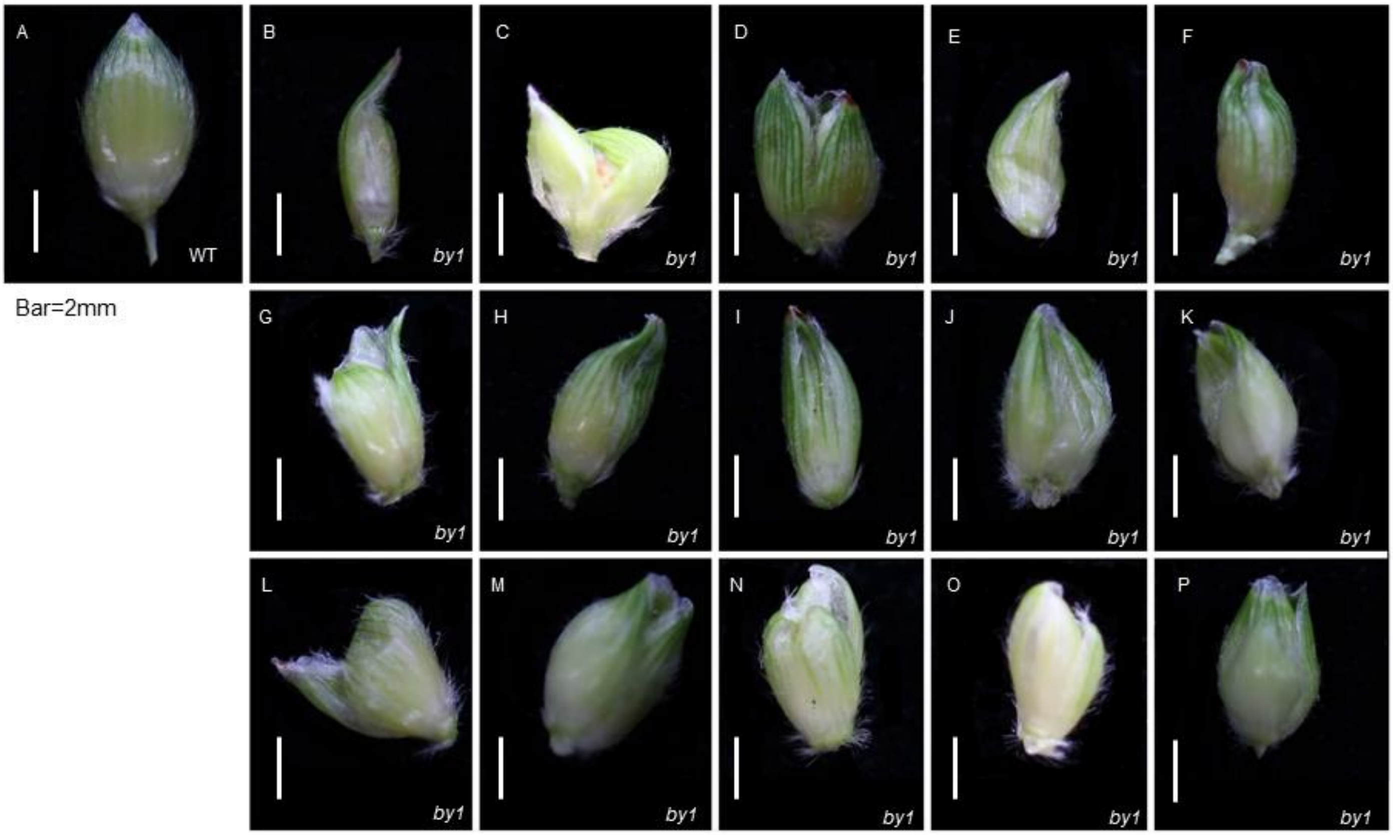

Supplemental Figure S4 Fertile floret phenotypes comparison between WT and *by1* mutant.

(A) The normal fertile floret phenotype in WT. The normal fertile floret of sorghum has a lemma and palea, both of which are membranous. Lemma larger, palea small and thin. There are 3 stamens and 1 pistil between palea and lemma. Bar=2 mm.

(B-P) Various abnormal fertile floret phenotypes in *by1* mutant. Lemma or palea or both are abnormal compared to that of WT. Bar=2 mm.

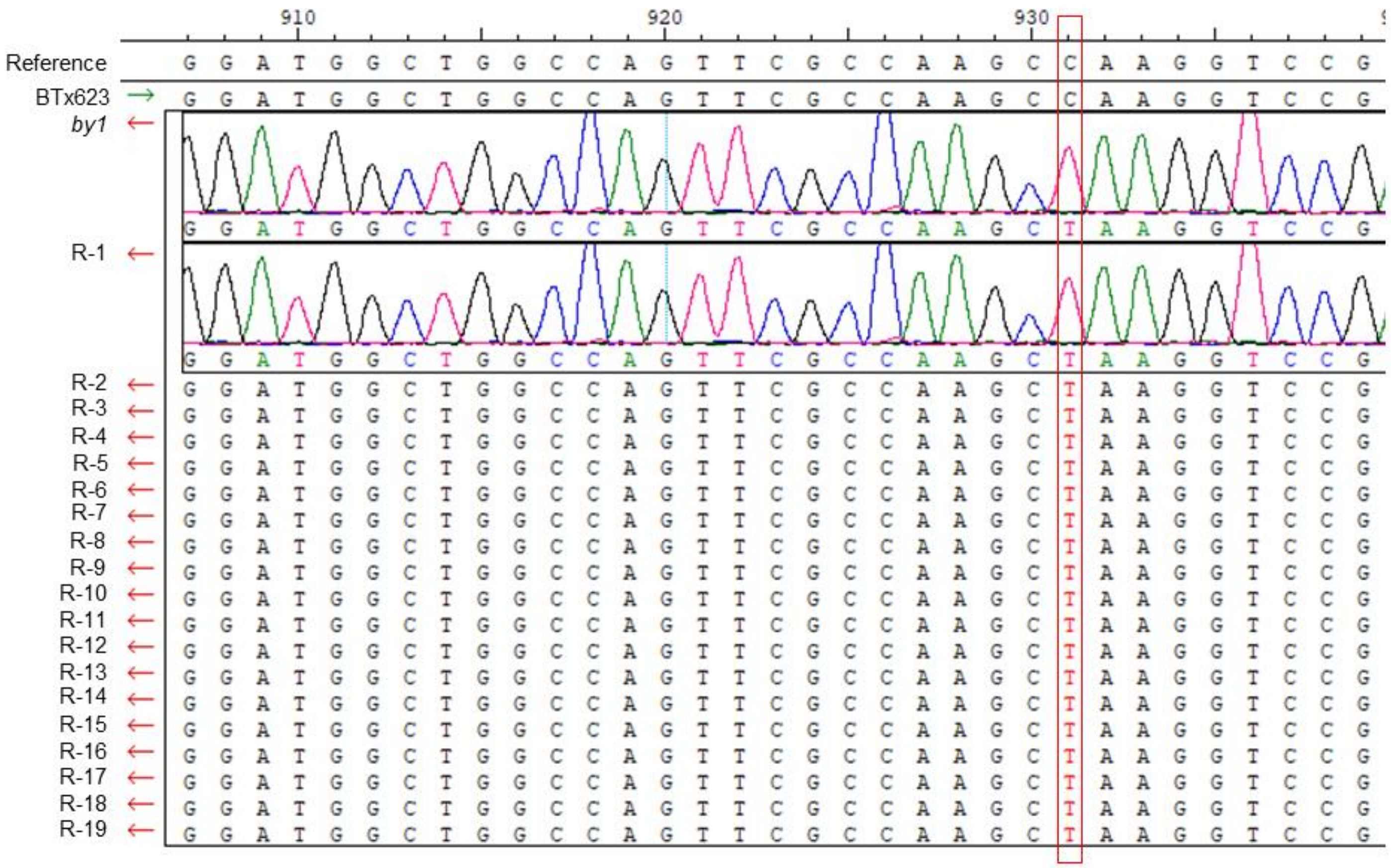

Supplemental Figure S5 Sequencing and alignment of the mutation site among recessive individual plants (R), *by1* and WT (BTx623).

BTx623 represents *BY1* (wild-type) genotype.

by1 represents mutant genotype.

R represents recombinant recessive plants in Shangzhuang x *by1* F_2 population.

Red box indicates the mutation site in *by1* mutant.

A

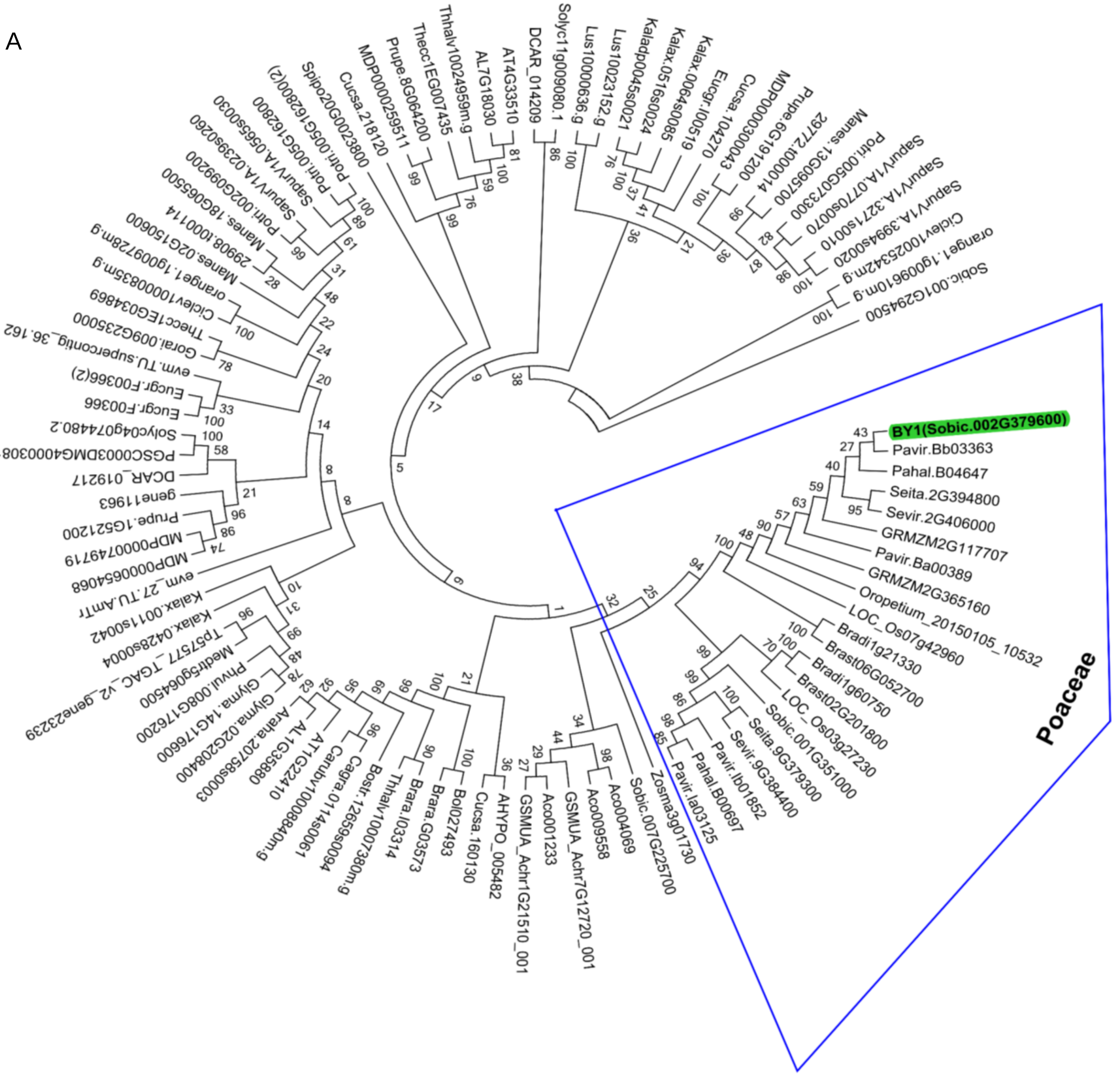

Sobic.001G294500
orange1.1g009610m.g
Ciclev10025342m.g
SapurV1A.3994s0020
SapurV1A.3271s0010
SapurV1A.0770s0070
Potri.005G073300
Manes.13G095700
29772.t000014
Prupe.6G191200
MDP0000300043
Cucsa.104270
Eucgr.I00519
Kalax.0064s0085
Kalax.0516s0024
Kaladp0045s0021
Lus10023152.g
Lus10000636.g
Solyc11g009080.1
DCAR_014209
AT4G33510
AL7G18030
Thhalv10024959m.g
Thecc1EG007435
Prupe.8G064200
MDP0000259511
Cucsa.218120
Spipo20G0023800
Potri.005G162800(2)
Potri.005G162800
SapurV1A.0565s0030
SapurV1A.0239s0260
Potri.002G099200
Manes.18G065500
29908.t000114
Manes.02G150600
orange1.1g009728m.g
Ciclev10000835m.g
Thecc1EG034869
Gorai.009G235000
evm.TU.supercontig_36.162
Eucgr.F00366(2)
Eucgr.F00366
Solyc04g074480.2
PGSC0003DMG400030812
DCAR_019217
gene11963
Prupe.1G521200
MDP0000749719
MDP0000654068
evm_27.TU.AmTr
Kalax.0011s0042
Kalax.0428s0004
Tp57577_TGAC_v2_gene23239
Medtr5g064500
Phvul.008G176200
Glyma.14G176600
Glyma.02G208400
Araha.20758s0003
AL1G35880
AT1G22410
Carubv10008840m.g
Cagra.0114s0061
Bostr.12659s0094
Thhalv10007380m.g
Brara.I03314
Brara.G03573
Bol027493
Cucsa.160130
AHYPO_005482
GSMUA_Achr1G21510_001
Aco001233
GSMUA_Achr7G12720_001
Aco009558
Aco004069
Sobic.007G225700
Zosma3g01730
Pavir.Ia03125
Pahal.B00697
Pavir.Ib01852
Sevir.9G384400
Seita.9G379300
Sobic.001G351000
LOC_Os03g27230
Brast02G201800
Bradi1g60750
Brast06G052700
Bradi1g21330
LOC_Os07g42960
Oropetium_20150105_10532
GRMZM2G365160
Pavir.Ba00389
GRMZM2G117707
Sevir.2G406000
Seita.2G394800
Pahal.B04647
Pavir.Bb03363
BY1(Sobic.002G379600)
Poaceae

B

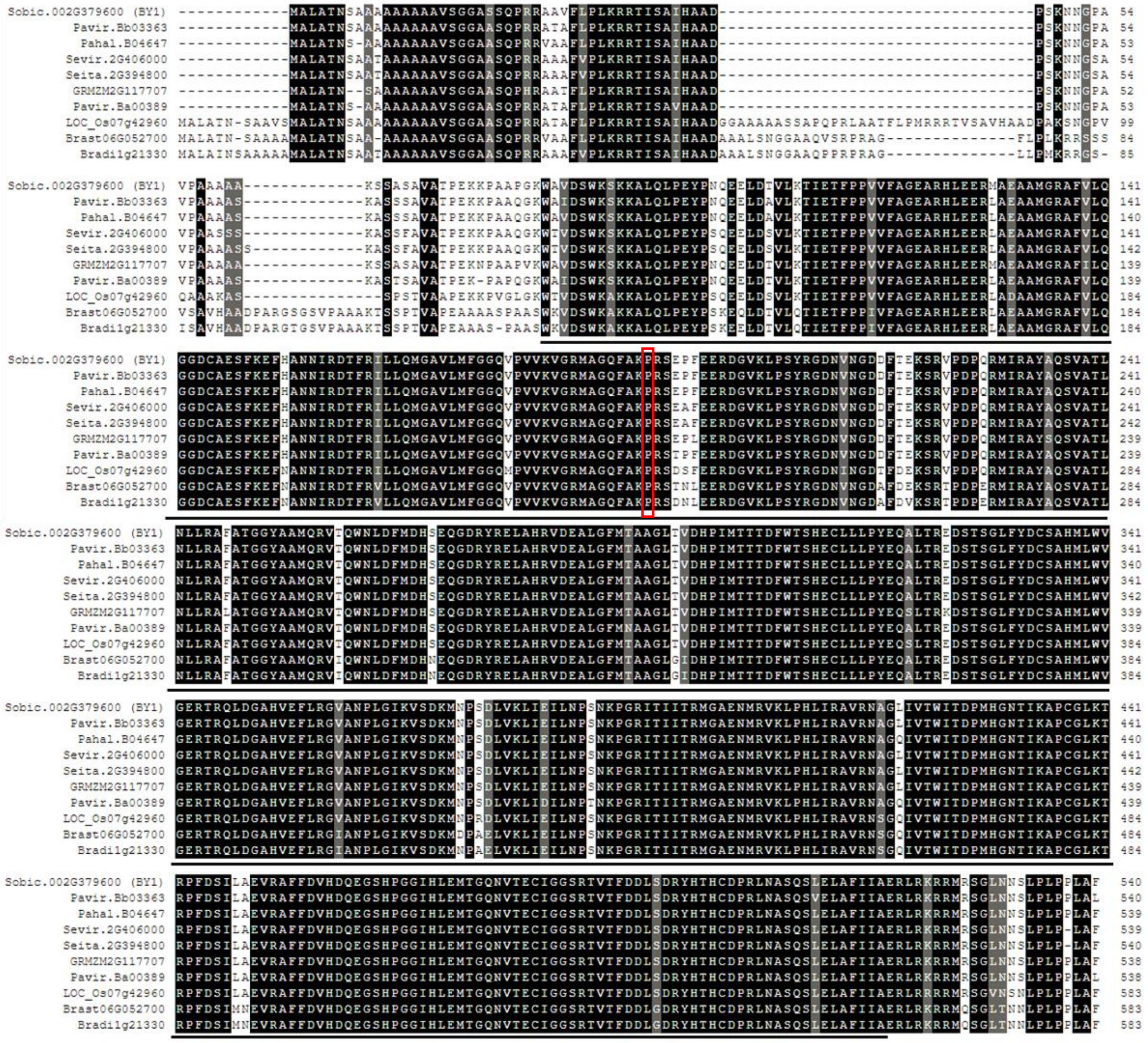

Supplemental Figure S6 Phylogenetic analysis of *BY1* and homologs.

(A) MEGA 5.0 was used to construct the phylogenetic tree by using the neighbor-joining method. Numbers above the branches represent bootstrap support based on 1000 bootstrap replicates. Branch length represents substitutions per site.

(B) Amino acid sequence alignment of poaceae closest to *BY1*. The black underline indicates the functional domain of *BY1*. Red box indicates the mutation site in *by1*.

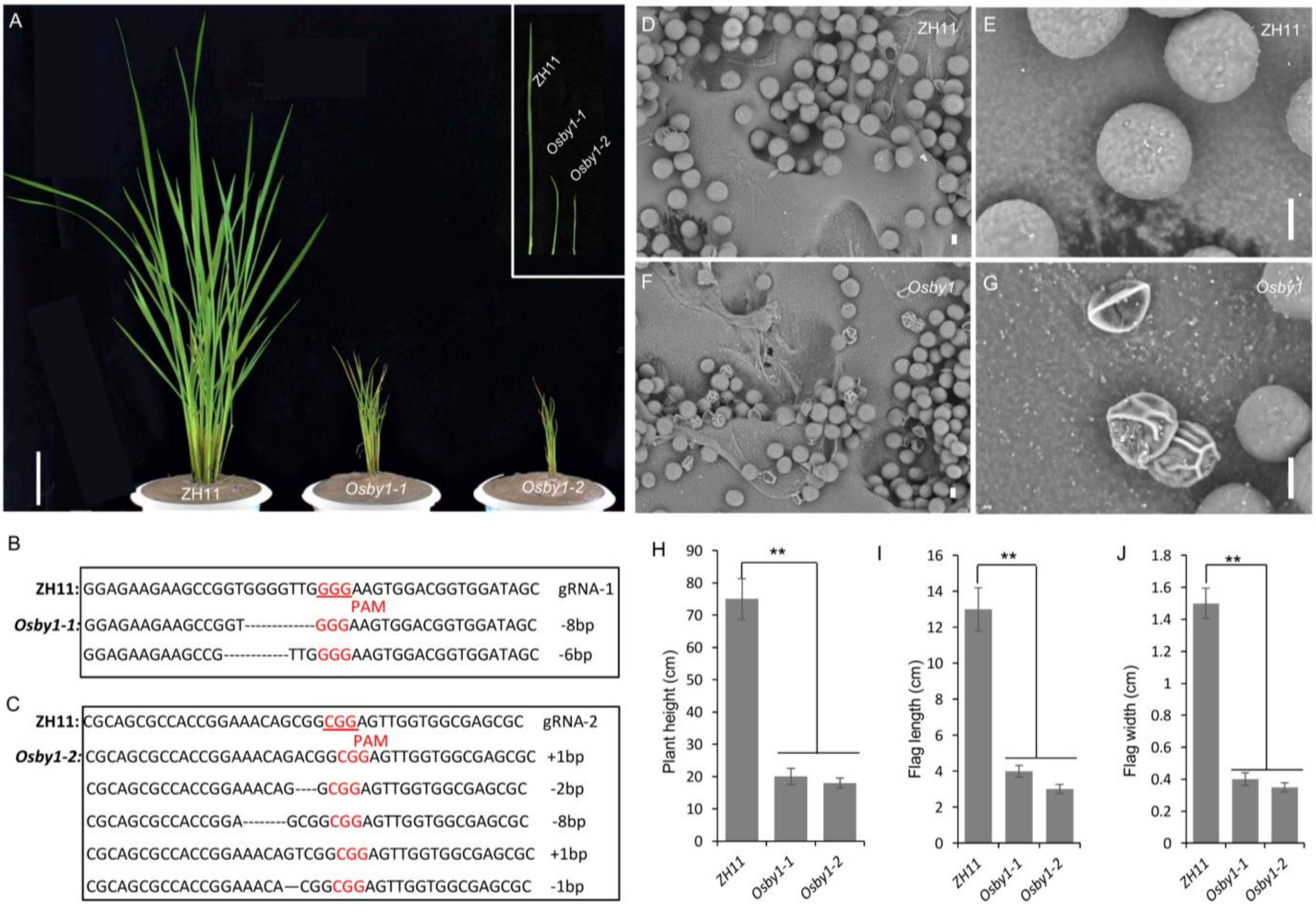

Supplemental Figure S7 Phenotypic comparison between ZH11 and edited positive plants.

(A) Phenotypes of ZH11and transgenic positive plants. Bar=10 cm.

(B) Two different genotypes produced by target gRNA-1 (*Osby1-1*). Red font indicates the PAM sequence of gRNA-1.

(C) Five different genotypes produced by target gRNA-2 (*Osby1-2*). Red font indicates the PAM sequence of gRNA-2.

(D-G) Pollen morphology comparison between ZH11 and *Osby1*. Bar=20 μm.

(H-J) Phenotypic comparison of agronomic characters between ZH11 and transgenic positive plants. [Values are mean ± SD (n=10). Two-tailed Student's *t*-test was performed between WT and *Osby1* (*P <0.05, **P <0.01)]

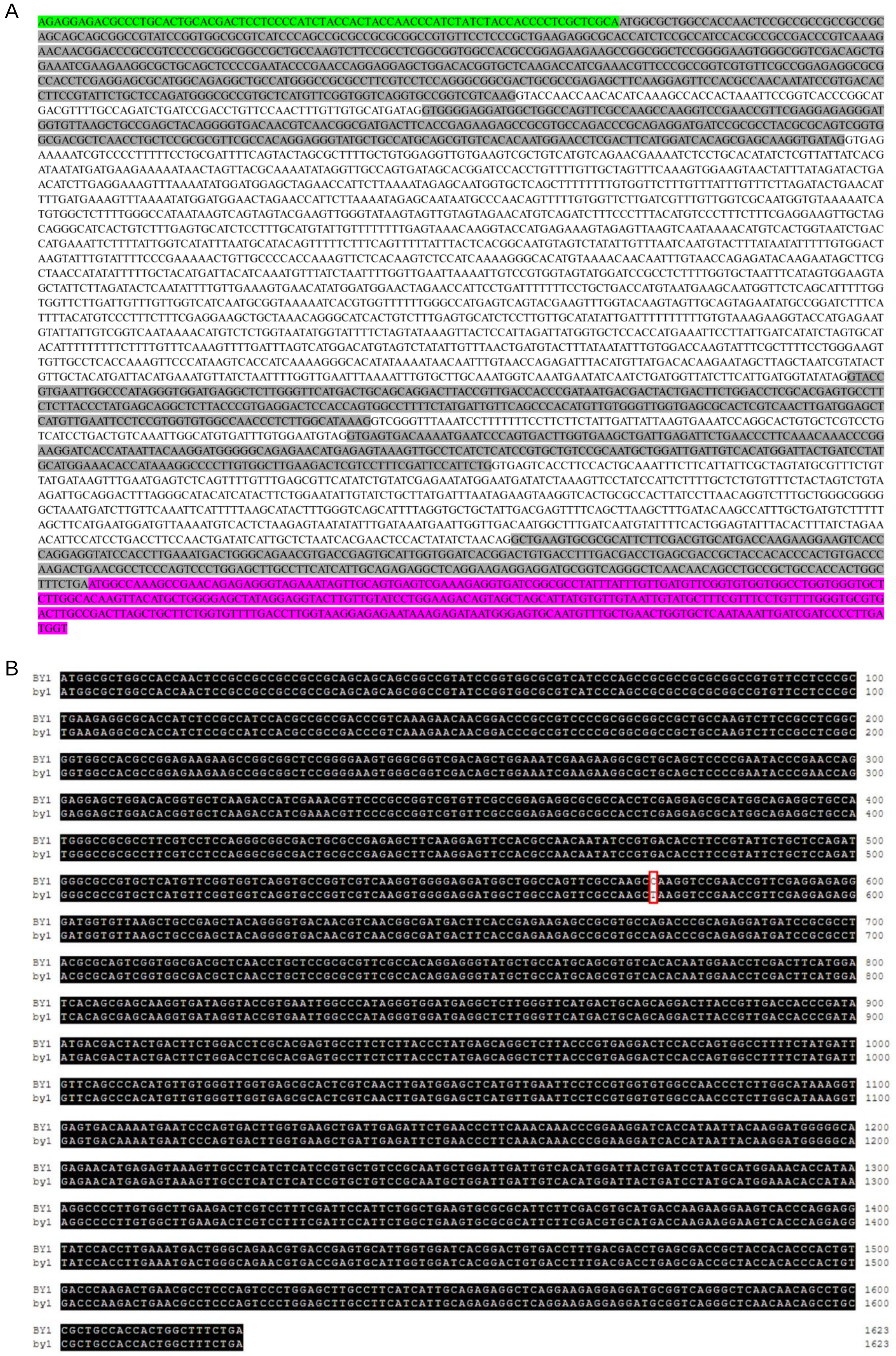
A
B
BY1
by1

C

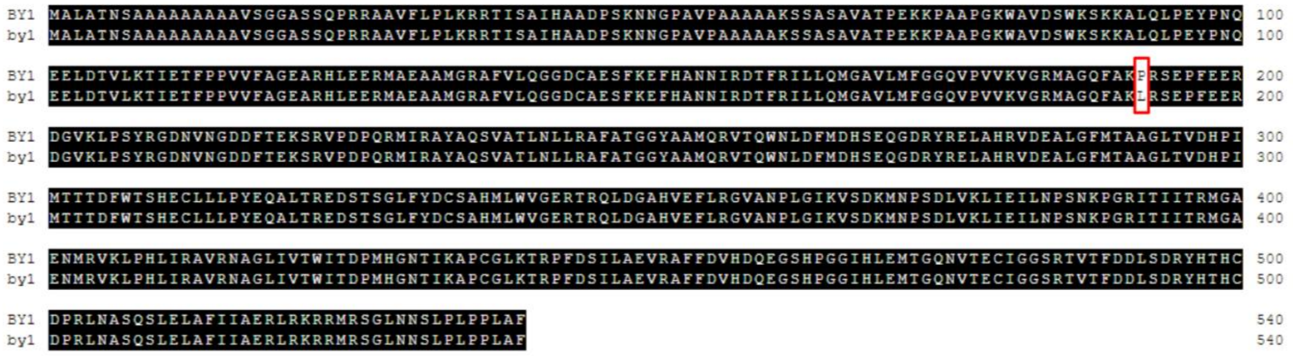

Supplemental Figure S8 Sequence information of *BY1*.

(A) The sequences marked with green and purple represent 5'UTR and 3'UTR respectively, the sequences marked with gray represent exons, and the sequences without any color represent introns.

(B) CDS sequence alignment between WT and *by-1* mutant. Red box is the mutation site.

(C) Amino acid sequence alignment between WT and *by-1* mutant. Red box is the mutation site.

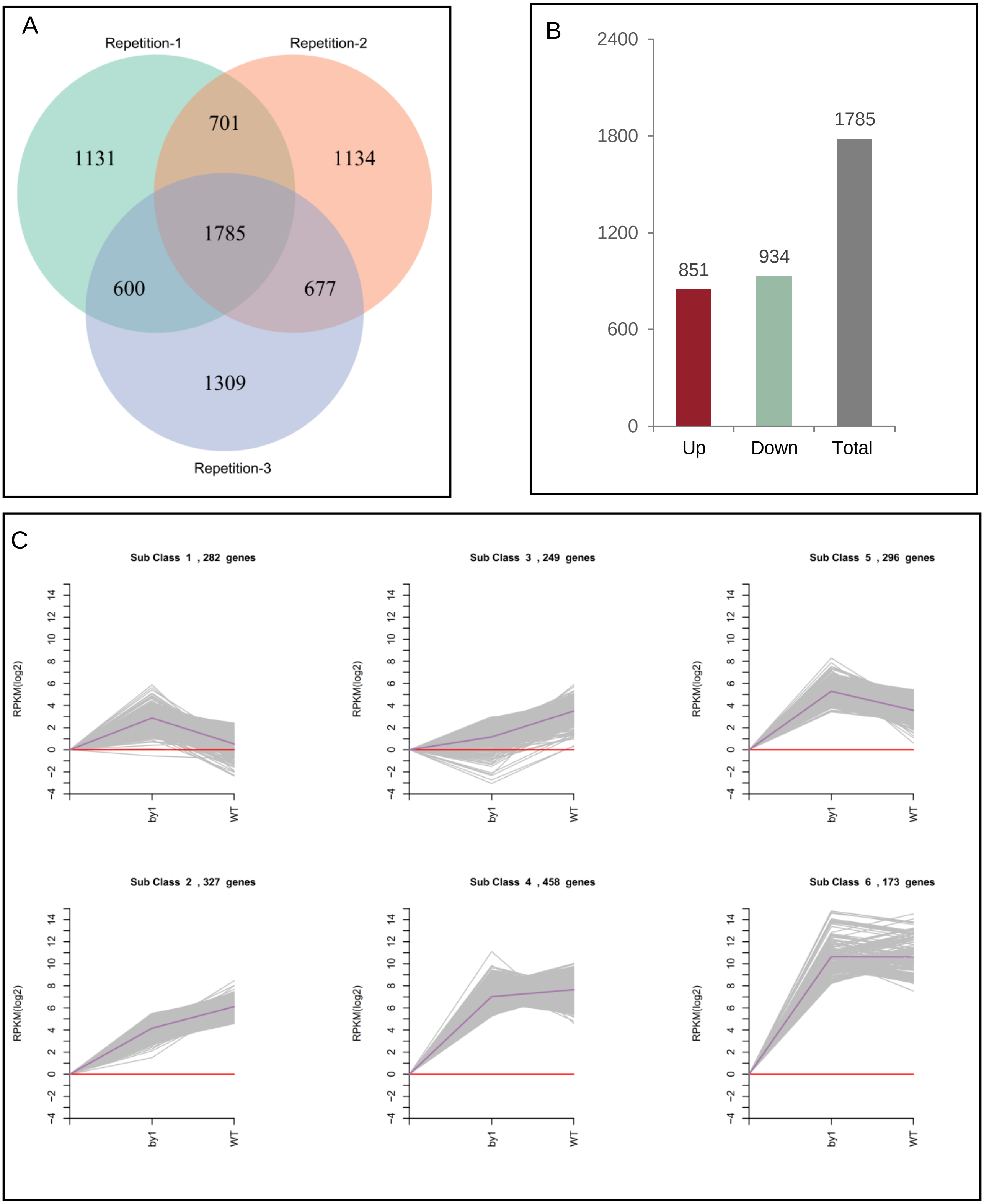
A
Repetition-1
Repetition-2
Repetition-3
1131
701
1134
1785
600
677
1309
B
2400
1800
1200
600
0
1785
851
934
Up
Down
Total
C
Sub Class 1 , 282 genes
Sub Class 3 , 249 genes
Sub Class 5 , 296 genes
Sub Class 2 , 327 genes
Sub Class 4 , 458 genes
Sub Class 6 , 173 genes
RPKM(log2)
by1
WT

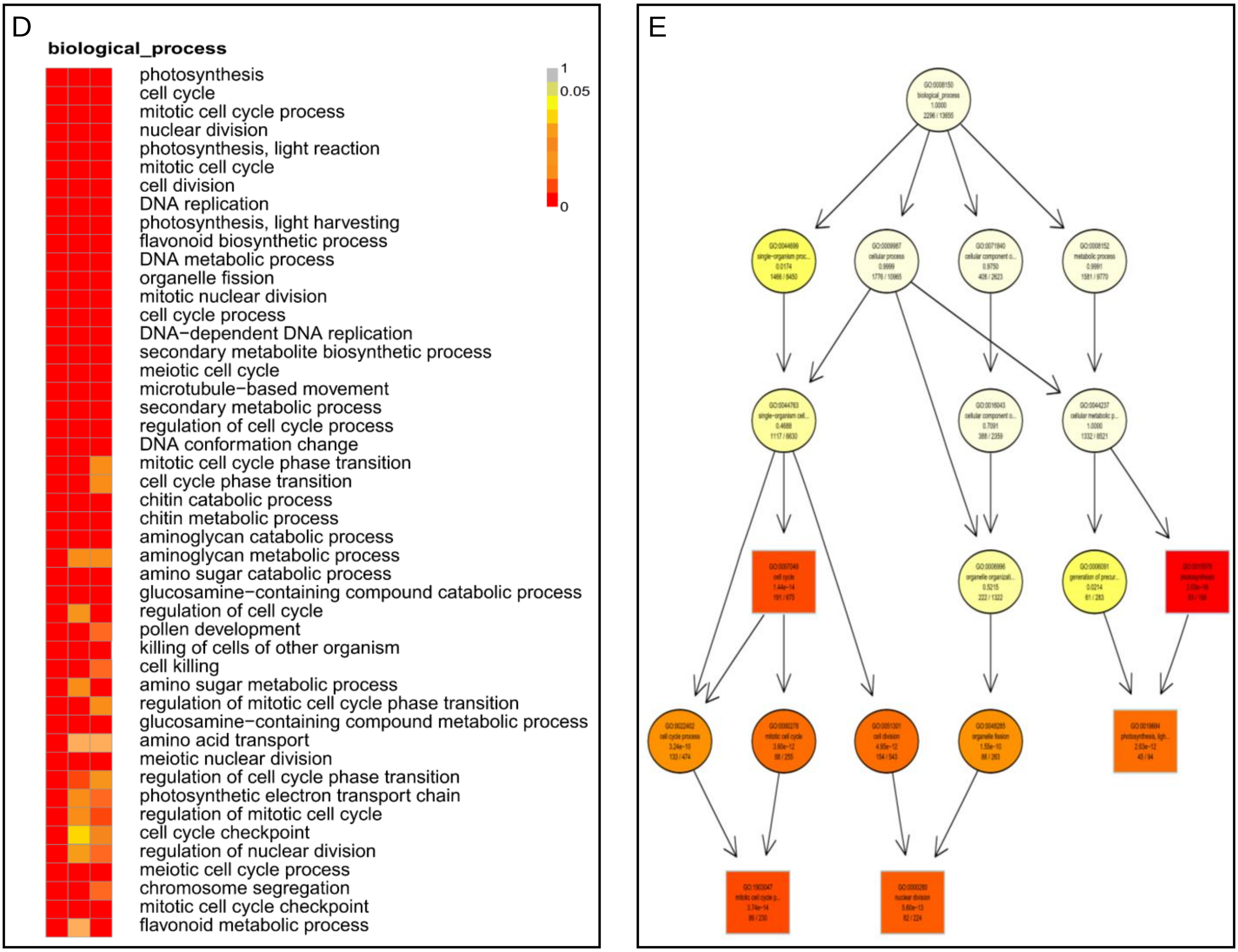

Supplemental Figure S9 Transcriptome analysis information of *BY1*.

(A) The total number of different expression genes (DEGs) in three repetitions.

(B) The number of up-regulated and down regulated DEGs.

(C) The number of DEGs in different subclasses.

(D, E) DEG clusters in different biological pathways.

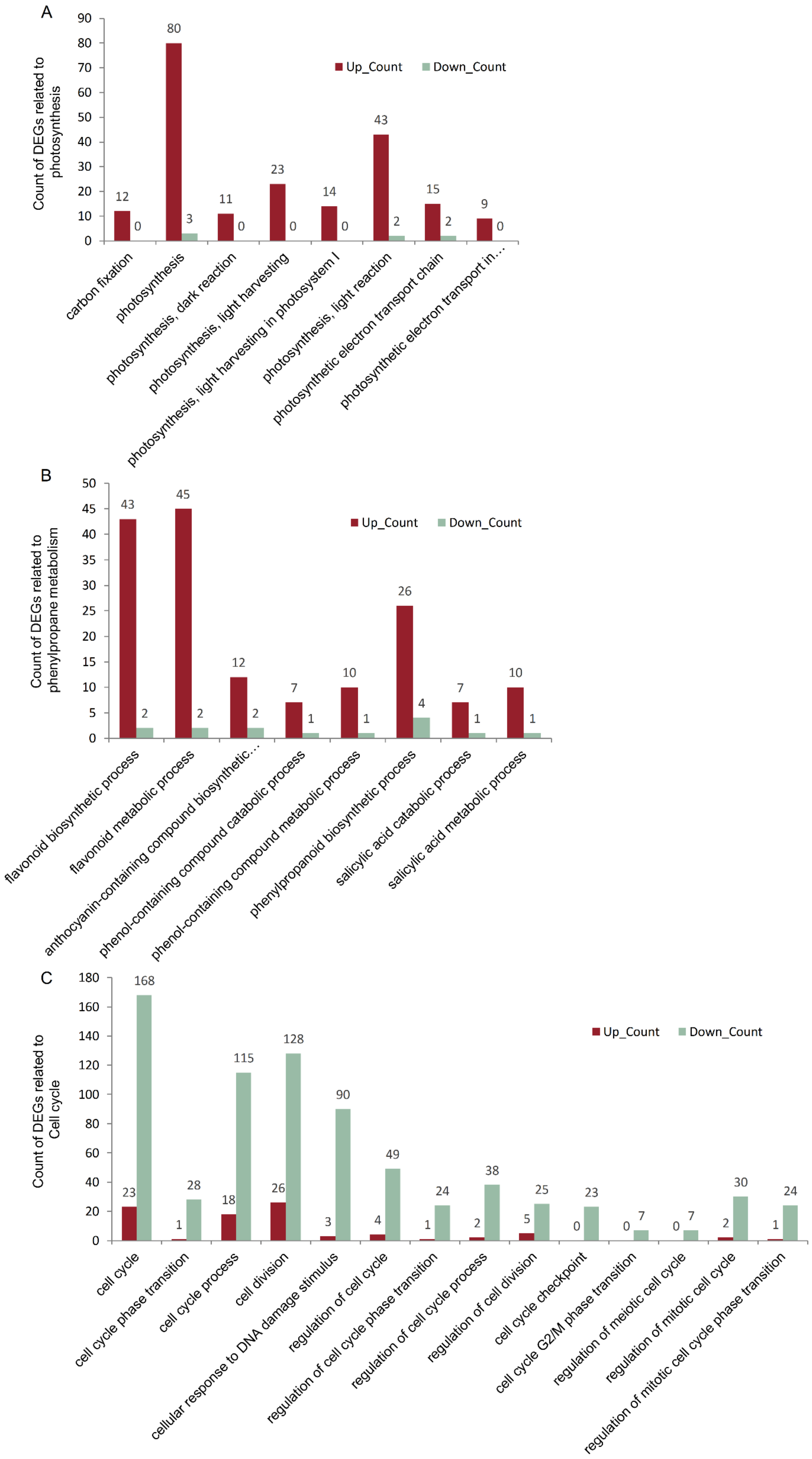
A
Count of DEGs related to photosynthesis
Up_Count
Down_Count
12
0
80
3
11
0
23
0
14
0
43
2
15
2
9
0
carbon fixation
photosynthesis
photosynthesis, dark reaction
photosynthesis, light harvesting
photosynthesis, light harvesting in photosystem I
photosynthesis, light reaction
photosynthetic electron transport chain
photosynthetic electron transport in…
B
Count of DEGs related to phenylpropane metabolism
Up_Count
Down_Count
43
2
45
2
12
2
7
1
10
1
26
4
7
1
10
1
flavonoid biosynthetic process
flavonoid metabolic process
anthocyanin-containing compound biosynthetic…
phenol-containing compound catabolic process
phenol-containing compound metabolic process
phenylpropanoid biosynthetic process
salicylic acid catabolic process
salicylic acid metabolic process
C
Count of DEGs related to Cell cycle
Up_Count
Down_Count
23
168
1
28
18
115
26
128
3
90
4
49
1
24
2
38
5
25
0
23
0
7
0
7
2
30
1
24
cell cycle
cell cycle phase transition
cell cycle process
cell division
cellular response to DNA damage stimulus
regulation of cell cycle
regulation of cell cycle phase transition
regulation of cell cycle process
regulation of cell division
cell cycle checkpoint
cell cycle G2/M phase transition
regulation of meiotic cell cycle
regulation of mitotic cell cycle
regulation of mitotic cell cycle phase transition

Supplemental Figure S10 DEGs information related to photosynthesis, phenylpropane metabolism and cell cycle processes.

(A) DEGs information related to photosynthesis.

(B) DEGs information related to phenylpropane metabolism.

(C) DEGs information related to cell cycle processes.

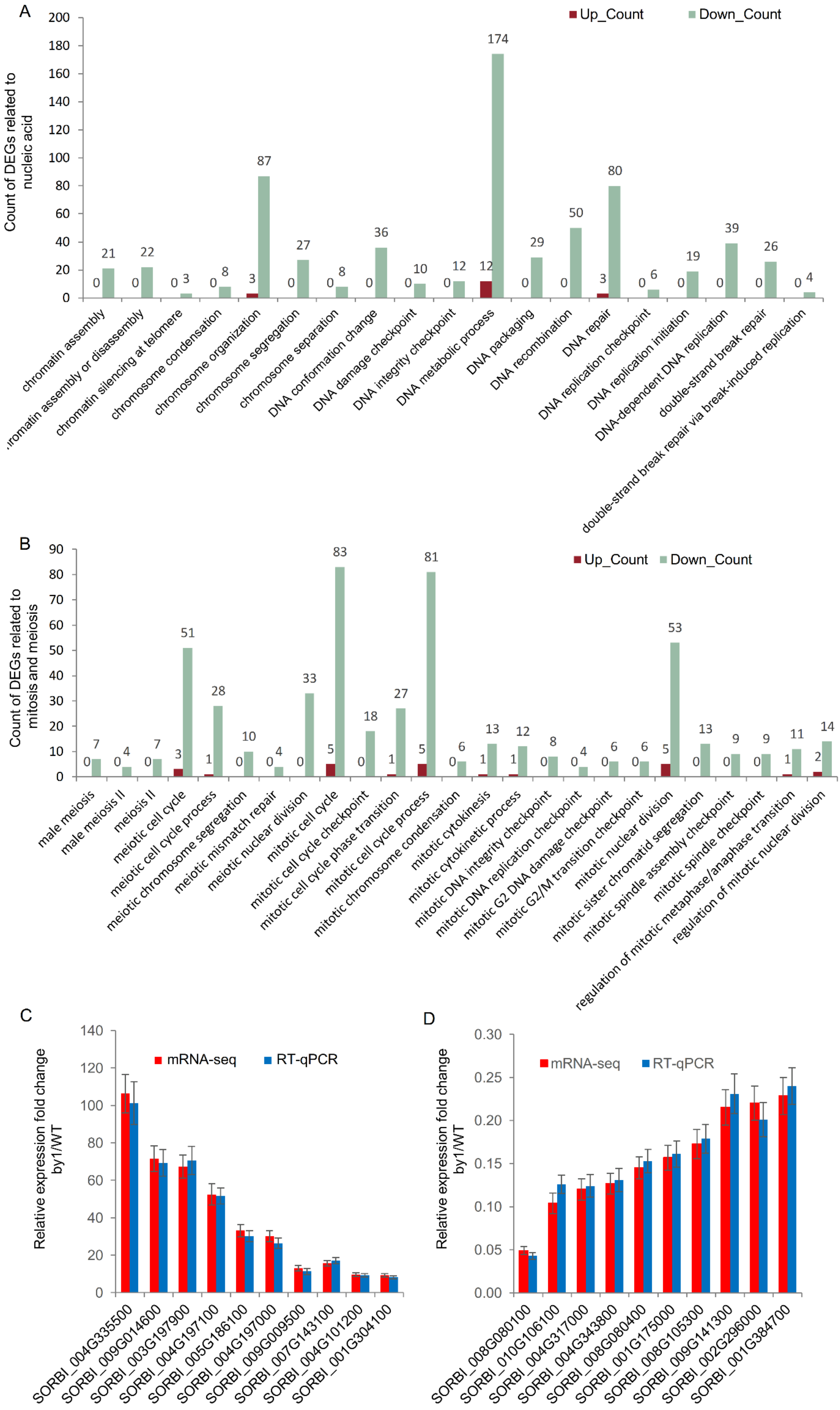
A
Up_Count
Down_Count
Count of DEGs related to nucleic acid
chromatin assembly
chromatin assembly or disassembly
chromatin silencing at telomere
chromosome condensation
chromosome organization
chromosome segregation
chromosome separation
DNA conformation change
DNA damage checkpoint
DNA integrity checkpoint
DNA metabolic process
DNA packaging
DNA recombination
DNA repair
DNA replication checkpoint
DNA replication initiation
DNA-dependent DNA replication
double-strand break repair
double-strand break repair via break-induced replication
B
Up_Count
Down_Count
Count of DEGs related to mitosis and meiosis
male meiosis
male meiosis II
meiosis II
meiotic cell cycle
meiotic cell cycle process
meiotic chromosome segregation
meiotic mismatch repair
meiotic nuclear division
mitotic cell cycle
mitotic cell cycle checkpoint
mitotic cell cycle phase transition
mitotic cell cycle process
mitotic chromosome condensation
mitotic cytokinesis
mitotic cytokinetic process
mitotic DNA integrity checkpoint
mitotic DNA replication checkpoint
mitotic G2 DNA damage checkpoint
mitotic G2/M transition checkpoint
mitotic nuclear division
mitotic sister chromatid segregation
mitotic spindle assembly checkpoint
mitotic spindle checkpoint
regulation of mitotic metaphase/anaphase transition
regulation of mitotic nuclear division
C
mRNA-seq
RT-qPCR
Relative expression fold change by1/WT
SORBI_004G335500
SORBI_009G014600
SORBI_003G197900
SORBI_004G197100
SORBI_005G186100
SORBI_004G197000
SORBI_009G009500
SORBI_007G143100
SORBI_004G101200
SORBI_001G304100
D
mRNA-seq
RT-qPCR
Relative expression fold change by1/WT
SORBI_008G080100
SORBI_010G106100
SORBI_004G317000
SORBI_004G343800
SORBI_008G080400
SORBI_001G175000
SORBI_008G105300
SORBI_009G141300
SORBI_002G296000
SORBI_001G384700

Supplemental Figure S11 DEGs information related to nucleic acid, mitosis and meiosis processes and the qRT-PCR verification.

(A) DEGs information related to nucleic acid.

(B) DEGs information related to mitosis and meiosis.

(C, D) Verification of the reliability of transcriptome data by RT-qPCR.

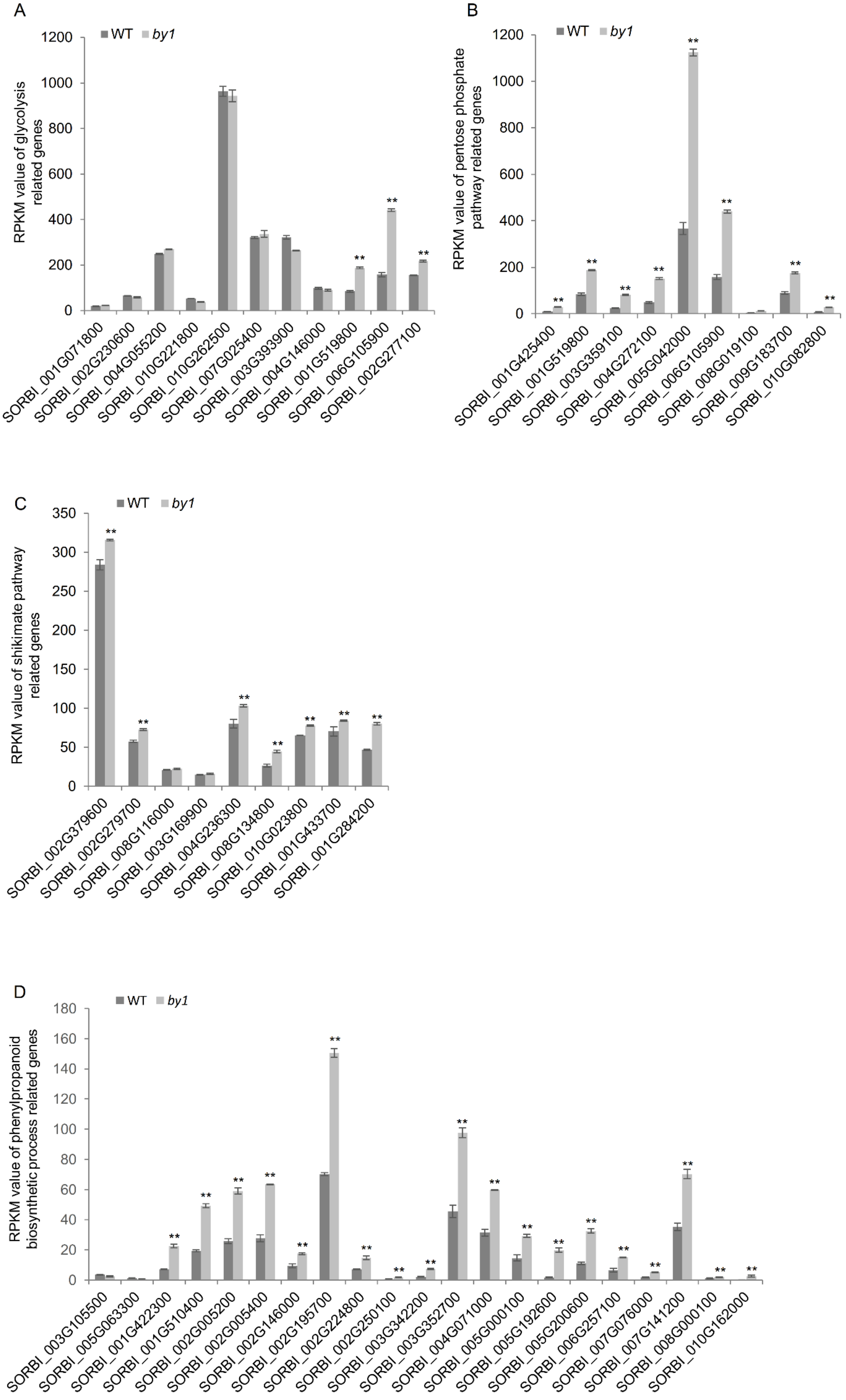

A
WT
by1
RPKM value of glycolysis related genes
0
200
400
600
800
1000
1200
**
SORBI_001G071800
SORBI_002G230600
SORBI_004G055200
SORBI_010G221800
SORBI_010G262500
SORBI_007G025400
SORBI_003G393900
SORBI_004G146000
SORBI_001G519800
SORBI_006G105900
SORBI_002G277100
B
WT
by1
RPKM value of pentose phosphate pathway related genes
0
200
400
600
800
1000
1200
**
SORBI_001G425400
SORBI_001G519800
SORBI_003G359100
SORBI_004G272100
SORBI_005G042000
SORBI_006G105900
SORBI_008G019100
SORBI_009G183700
SORBI_010G082800
C
WT
by1
RPKM value of shikimate pathway related genes
0
50
100
150
200
250
300
350
**
SORBI_002G379600
SORBI_002G279700
SORBI_008G116000
SORBI_003G169900
SORBI_004G236300
SORBI_008G134800
SORBI_010G023800
SORBI_001G433700
SORBI_001G284200
D
WT
by1
RPKM value of phenylpropanoid biosynthetic process related genes
0
20
40
60
80
100
120
140
160
180
**
SORBI_003G105500
SORBI_005G063300
SORBI_001G422300
SORBI_001G510400
SORBI_002G005200
SORBI_002G005400
SORBI_002G146000
SORBI_002G195700
SORBI_002G224800
SORBI_002G250100
SORBI_003G342200
SORBI_003G352700
SORBI_004G071000
SORBI_005G000100
SORBI_005G192600
SORBI_005G200600
SORBI_006G257100
SORBI_007G076000
SORBI_007G141200
SORBI_008G000100
SORBI_010G162000

Supplemental Figure S12 RPKM values related to glycolysis, pentose phosphate pathway, shikimate pathway and phenylpropanoid pathway genes.

(A) RPKM values related to glycolysis genes.

(B) RPKM values related to pentose phosphate pathway genes.

(C) RPKM values related shikimate pathway genes.

(D) RPKM values related phenylpropanoid biosynthetic process genes.

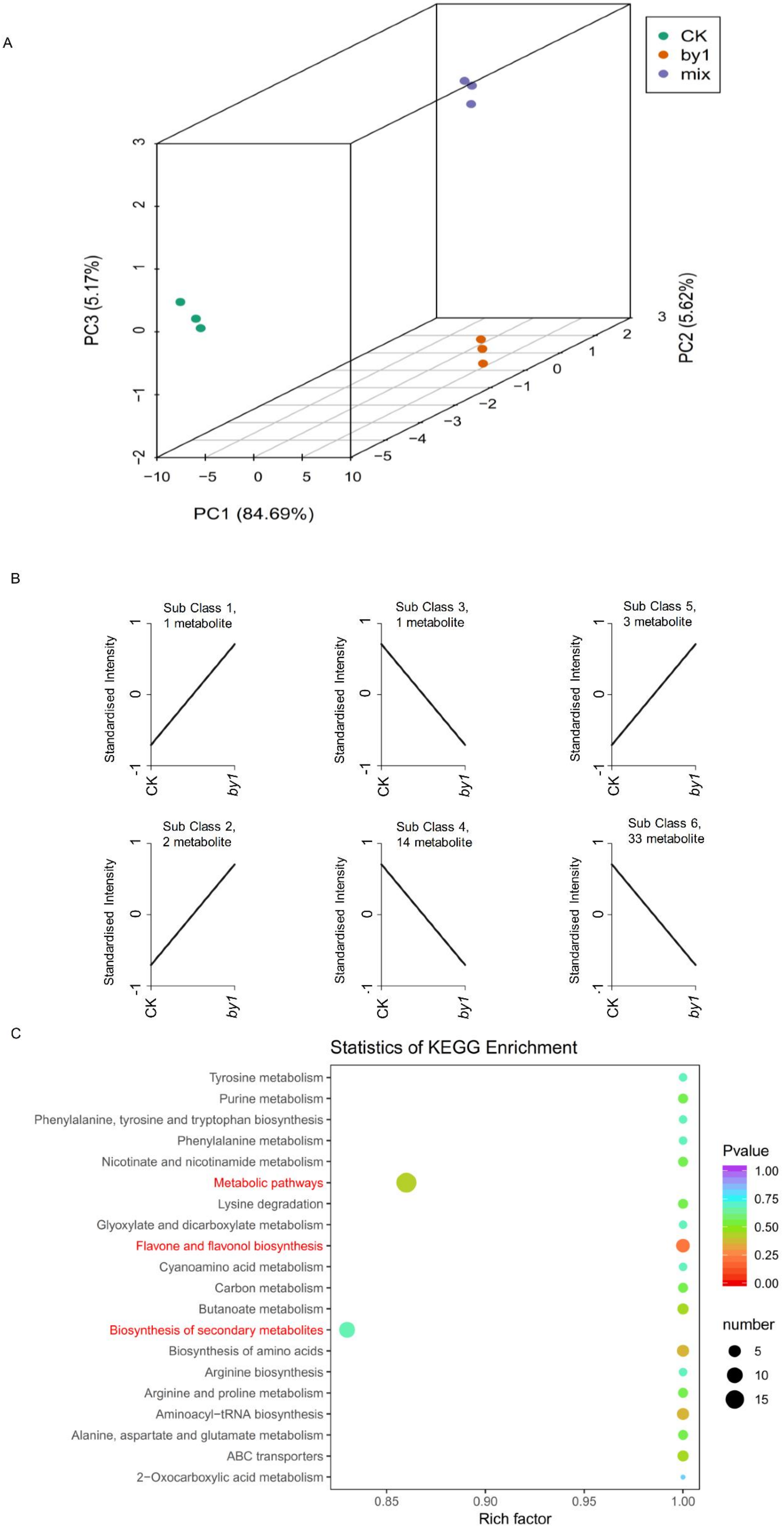
A
CK
by1
mix
PC3 (5.17%)
PC2 (5.62%)
PC1 (84.69%)
B
Sub Class 1, 1 metabolite
Sub Class 3, 1 metabolite
Sub Class 5, 3 metabolite
Sub Class 2, 2 metabolite
Sub Class 4, 14 metabolite
Sub Class 6, 33 metabolite
Standardised Intensity
CK
by1
C
Statistics of KEGG Enrichment
Tyrosine metabolism
Purine metabolism
Phenylalanine, tyrosine and tryptophan biosynthesis
Phenylalanine metabolism
Nicotinate and nicotinamide metabolism
Metabolic pathways
Lysine degradation
Glyoxylate and dicarboxylate metabolism
Flavone and flavonol biosynthesis
Cyanoamino acid metabolism
Carbon metabolism
Butanoate metabolism
Biosynthesis of secondary metabolites
Biosynthesis of amino acids
Arginine biosynthesis
Arginine and proline metabolism
Aminoacyl−tRNA biosynthesis
Alanine, aspartate and glutamate metabolism
ABC transporters
2−Oxocarboxylic acid metabolism
Rich factor
Pvalue
number

Supplemental Figure S13 Metabolic profiling information of *BY1*.

(A) The principal component analysis (PCA).

(B) The number of metabolites with significantly different content (MSDCs) in different subclasses.

(C) The KEGG enrichment of MSDCs.

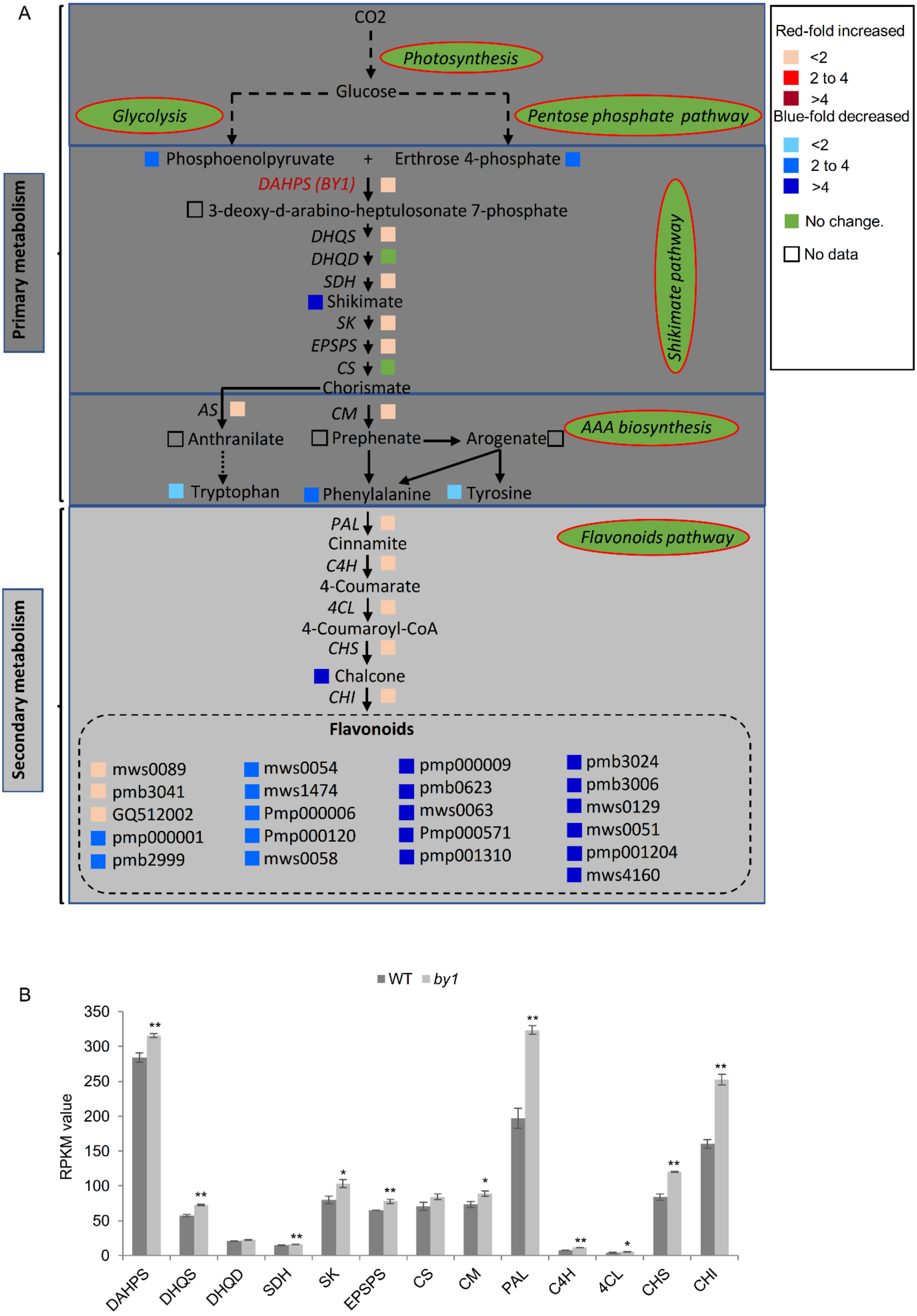
A
CO2
Photosynthesis
Glucose
Glycolysis
Pentose phosphate pathway
Primary metabolism
Phosphoenolpyruvate + Erthrose 4-phosphate
DAHPS (BY1)
3-deoxy-d-arabino-heptulosonate 7-phosphate
DHQS
DHQD
SDH
Shikimate
SK
EPSPS
CS
Chorismate
Shikimate pathway
AS
CM
Anthranilate
Prephenate
Arogenate
AAA biosynthesis
Tryptophan
Phenylalanine
Tyrosine
Secondary metabolism
PAL
Cinnamite
C4H
4-Coumarate
4CL
4-Coumaroyl-CoA
CHS
Chalcone
CHI
Flavonoids pathway
Flavonoids
mws0089
pmb3041
GQ512002
pmp000001
pmb2999
mws0054
mws1474
Pmp000006
Pmp000120
mws0058
pmp000009
pmb0623
mws0063
Pmp000571
pmp001310
pmb3024
pmb3006
mws0129
mws0051
pmp001204
mws4160
Red-fold increased
<2
2 to 4
>4
Blue-fold decreased
<2
2 to 4
>4
No change.
No data
B
WT by1
RPKM value
350
300
250
200
150
100
50
0
DAHPS
DHQS
DHQD
SDH
SK
EPSPS
CS
CM
PAL
C4H
4CL
CHS
CHI

Supplemental Figure S14 A regulation metabolic map related to BY1.

(A) The serial numbers in the dotted box represent the different types of flavonoid metabolites shown in Table S2. *DAHPS*, 3-Deoxy-D-arabino-heptulosonate7-phosphate synthase; *DHQS*, 3-Dehydroquinate synthase; *DHQD*, 3-Dehydroquinate dehydratase; *SDH*, Shikimate dehydrogenase; *SK*, Shikimate kinase; *EPSPS*, 5-Enolpyruvylshikimate 3-phosphate synthase; *CS*, Chorismate synthase; *AS*, Anthranilate Synthase; *CM*, Chorismate mutase; *PAL*, phenylalanine ammonia-lyase; *C4H*, Cinnamate-4-hydroxylase; *4CL*, 4-coumaroyl: CoA-ligase; *CHS*, Chalcone synthase; *CHI*, Chalcone isomerase.

(B) RPKM value of genes related to shikimate pathway and its downstream pathways.

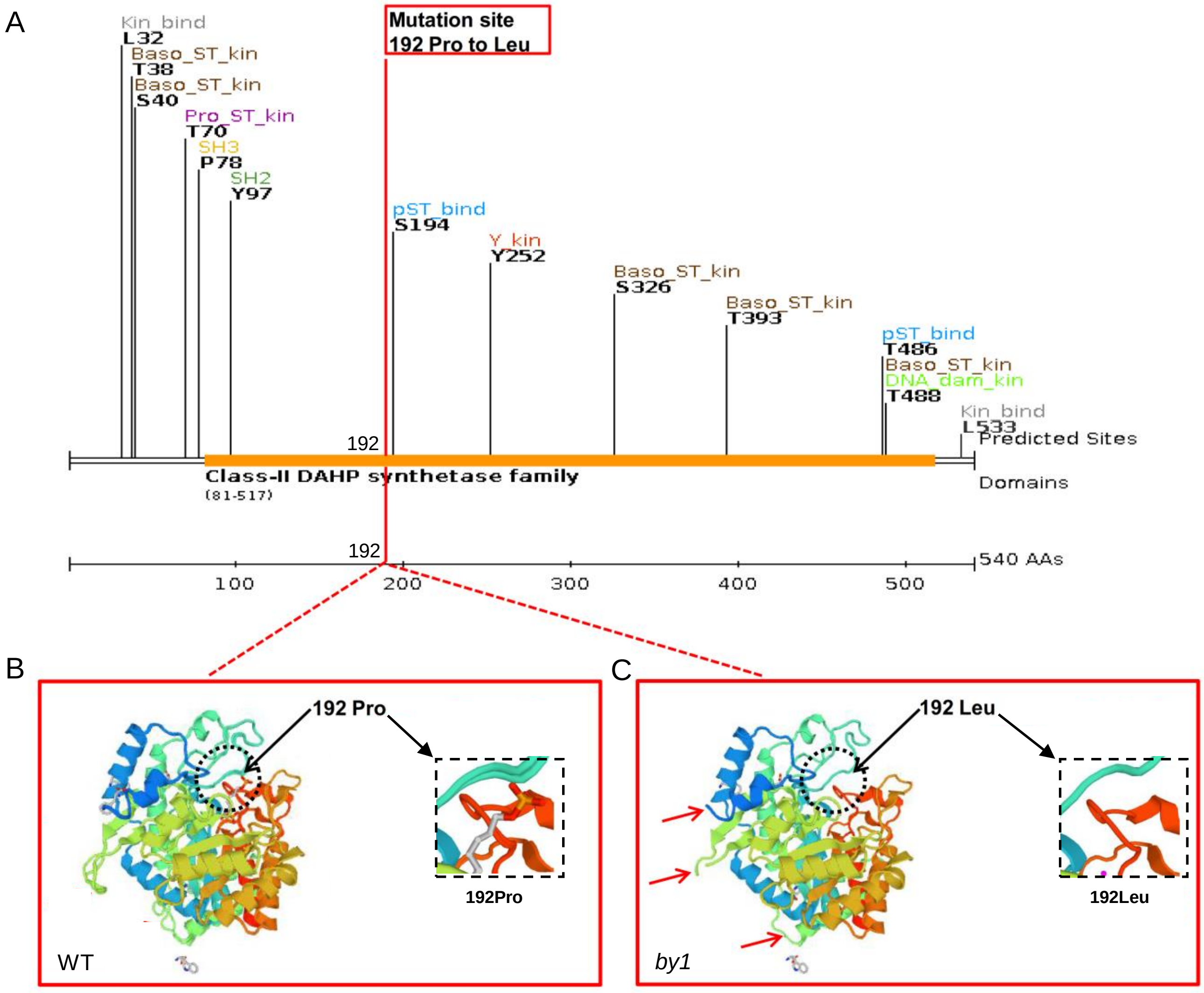

Supplemental Figure S15 Comparison of tertiary structure of BY1 protein in WT and *by1* mutant.

(A) Basic information of *BY1* including the number of amino acids, the functional domain and the mutation position of *by1* mutant (192nd amino acid substitution from Pro to Leu).

(B) The tertiary structure of BY1 protein in WT. Black arrows indicate the 192nd Pro site of *BY1* protein and the black dotted box shows the tertiary structure of this site.

(C) The tertiary structure of BY1 protein in *by1* mutant. Black arrows indicate the 192nd mutation (Pro→Leu) site in *by1* mutant and the black dotted box shows the tertiary structure difference of this site compared to that of WT. Red arrows represent other obvious protein structure alterations between WT and *by1* due to the conversion of the 192nd amino acid from Pro to Leu.
